# Supplementary material for: Exonic enhancers are a widespread class of dual-function regulatory elements
Source: Nat Commun. 2026 Apr 2;17:4755. doi: 10.1038/s41467-026-71220-6 (PMC13216554; doi:10.1038/s41467-026-71220-6)
Supplement: Supplementary file 1 — Supplementary Information [file 41467_2026_71220_MOESM1_ESM.pdf]

SUPPLEMENTARY INFORMATION

Exonic enhancers are a widespread class of dual-function regulatory elements

Jean-Christophe Mouren, Magali Torres, Antoinette van Ouwerkerk, Iris Manosalva, Frederic Gallardo, Salvatore Spicuglia, Benoit Ballester

Supplementary tables **2**

Table S1: Permutation-based enrichment of genomic and epigenomic features in coding exons and cEEs across four species. 2

Table S2: Population-genetic and evolutionary metrics for cEEs versus control coding exons. 4

Table S3: Allele-frequency distribution of gnomAD/TCGA SNVs in cEEs versus control exons. 5

Table S4: cEEs tested by reporter luciferase assay Figure 3a. 6

Table S5: Tested promoters on Figure 3bc. 6

Table S6: cEEs mutated on Figure 3de. 7

Table S7: gRNAs used for CRISPRi Figure 6b-e. 8

Table S8: Primers to assess gene expression Figure 6b-e, Supp Figure S28. 8

Supplementary figures **9**

Figure S1: Re-evaluation of exon enhancer candidates from Birnbaum et al. 9

Figure S2: Genomic distribution of ChIP-seq and DNase-seq data across four species. 10

Figure S3: Transcription factor occupancy in coding exons across species. 11

Figure S4: Genomic distribution of transcription factor binding across exon classes. 12

Figure S5: Transcription factor binding density in internal exons. 13

Figure S6: Position of TF-binding summits along cEEs in four species. 14

Figure S7: Enrichment of transcription factors in cEEs. 15

Figure S8: Prevalence of cEEs across gene transcript isoforms. 16

Figure S9 : Constitutive inclusion of cEEs across GTEx tissues. 17

Figure S10 : APPRIS isoform annotation of cEEs versus control exons. 18

Figure S11: Chromatin accessibility at exon enhancers across species. 19

Figure S12: Enrichment of histone modifications at exon enhancers. 20

Figure S13: ENCODE STARR-seq activity in exonic enhancers stratified by biotype. 21

Figure S14: cEEs size and transcription factor binding site density. 22

Figure S15: GC content and CpG enrichment at exon enhancers. 23

Figure S16: Synonymous-to-total variant ratio for cEEs versus matched coding exons. 24

Figure S17: Cell line specificity and selection refinement of exon enhancer activity in STARR-seq. 25

Figure S18: GTEx expression of cEE-associated genes. 26

Figure S19: GTEx heatmap of cEE biotype groups. 27

Figure S20: Independent tissue-specificity metrics (HPA/Uhlén and ENCODE). 29

Figure S21: Variant density in cEEs and its relationship to TF-binding load. 30

Figure S22: Allele frequency distribution across TF-binding deciles in cEEs. 31

Figure S23: Variant-effect bias is not explained by regression to the mean. 32

Figure S24: Interaction landscape of exon enhancers across cellular and tissue contexts. 33

Figure S25: PanCancerAtlas variants in cEEs and their impact on transcription factor binding. 34

Figure S26: PanCancerAtlas lollipop genomic track. 35

Figure S27: GWAS Catalog Variants in candidate Exonic Enhancers (cEEs). 36

Figure S28: CRISPRi Exon controls. 37

Figure S29: Comparative conservation of exonic and intergenic enhancers between human and mice. 38

Figure S30: Evolutionary conservation and structural properties of exonic enhancers. 39

Figure S31: Selection of exonic enhancers based on transcription factor summit density. 41

Figure S32: STARR-seq experimental design and validation of exonic enhancer activity. 42

Figure S33: Validation of CRISPRi-competent K-562 cells by inhibition of CD81 expression. 43

Figure S34: Distribution and regulatory impact of gnomAD SNPs in exonic enhancers. 44

References **45**

Supplementary tables

Table S1: Permutation-based enrichment of genomic and epigenomic features in coding exons and cEEs across four species.

Enrichment of genomic and epigenomic features in coding exons (all) and candidate exonic enhancers (cEEs). For each feature we report the observed percentage overlap, the mean expected overlap from GC-, length- and chromosome-matched permutations, the resulting fold-enrichment, and the empirical P-value. Statistical significance was assessed using a permutation-based empirical test, with two-tailed empirical P-values derived from GC-, length-, and chromosome-matched permutations. Values are shown for human (*H. sapiens*), mouse (*M. musculus*), fruit fly (*D. melanogaster)*, and Arabidopsis thaliana. As anticipated for compact genomes (fly, plant), the dynamic range is smaller, yet enrichments for cEEs remain the strongest within each species. See “Permutation-based enrichment tests” in Methods for details. The relatively modest (yet significant) enrichment observed in the TFBS randomization test can be partly attributed to the larger size of cEEs. As shown in Supplementary Fig. 14, cEEs are significantly longer than other coding exons, which naturally increases the number of elements that can be shuffled within them, as noted in the bedtools shuffle documentation.

Feature	Species	Observed %	Expected %	Fold	Empirical P-val
DHS (all coding exons)	Human	3.90	3.0841	1.26	0
	Mouse	3.70	4.5311	0.82	0
	Fly	4.20	28.2635	0.15	0
	Arabidopsis	9.60	43.5015	0.22	0
TF peaks (all coding exons)	Human	4.20	2.7441	1.53	0
	Mouse	3.30	3.9689	0.83	0
	Fly	10.80	18.2469	0.59	0
	Arabidopsis	21.80	27.7524	0.79	0
DHS (cEE only)	Human	0.5033	0.3881	1.30	0
	Mouse	0.5614	0.4605	1.22	0
	Fly	1.4186	12.0603	0.12	0
	Arabidopsis	2.1403	4.4608	0.48	0
TF peaks (cEE only)	Human	0.5420	0.4593	1.18	0
	Mouse	0.6755	0.5598	1.21	0
	Fly	4.3032	7.9053	0.54	0
	Arabidopsis	4.2059	3.6140	1.16	0
TFBS (cEE only)	Human	15.640	14.751	1.06	0
	Mouse	11.948	11.157	1.07	0

	Fly	44.406	43.343	1.02	0
	Arabidopsis	14.506	13.407	1.08	0
cEEs conservation	Human/Mouse	28.0	6,94	4.03	0
H3K27ac (GTEx, cEE)	Human	0.6391	0.6100	1.05	2×10^{-4}
H3K27ac (ENCODE, cEE)	Human	0.9026	0.8770	1.03	0
H3K4me1 (ENCODE, cEE)	Human	0.8847	0.8090	1.09	0
H3K4me2 (ENCODE, cEE)	Human	8.0721	1.0881	7.42	0
ATAC-seq (cEE)	Human	0.3692	1.0130	0.36	0
	Mouse	0.4582	1.4215	0.32	0
	Fly	1.4055	15.3990	0.82	0
	Arabidopsis	4.0319	4.9349	0.09	0

Table S2: Population-genetic and evolutionary metrics for cEEs versus control coding exons.

The table summarises eight indicators of selective constraint. The first reports short-termpolymorphis m in gnomAD[1] v3.1.2, the second one reports long-term divergence between human and mouse orthologues, and the last one combines the two data sources. We compared cEEsto contr ol exons (Ctrl–) with eight complementary population-genetic and evolutionary metrics. Forclarity w e summarize these metrics below:

Short-term polymorphism	Long-term divergence	Composite tests
• Median allele frequency (AF)<br>• Fraction of ultra-rare SNVs (AF ≤ 0.01 %)<br>• Synonymous SNVs per kb<br>• Synonymous / total variant ratio<br>• pN/pS (missense / synonymous)	• dN/dS in human–mouse orthologues	• Direction of Selection (DoS)<br>• Fixation Index (FI)

“Median AF” is the exon-level median variant allele frequency; lower values indicate rarer alleles. “Median synonymous SNVs per exon size” quantifies the density of putatively neutral changes. “Median synonymous / all ratio” is the fraction of synonymous variants per exon. pN/pS is the ratio of missense to synonymous counts in gnomAD; dN/dS is the ratio of fixed nonsynonymous to synonymous substitutions between human and mouse. The Direction-of-Selection statistic (DoS = dN/(dN + dS) – pN/(pN + pS)) and the Fixation Index (FI = (dN/dS)/(pN/pS)) compare divergence to polymorphism, with positive DoS or FI > 1 indicating an excess of fixed nonsynonymous changes. Two-sided Mann–Whitney tests compare per-exon distributions; Two-sided χ^2 tests compare pooled counts between cEEs (n = 13,481 exons; n = 870,139 variants) and Ctrl– exons (n = 13,481 exons; n = 236,177 variants).

cEEs have lower allele frequencies, more synonymous changes per base, a higher synonymous/total ratio, and a reduced pN/pS, consistent with stronger purifying selection in present-day humans. dN/dS is modestly higher for cEEs, and both DoS and FI are positive, suggesting that compensatory or adaptive amino-acid substitutions have accrued over longer evolutionary timescales while regulatory information embedded in cEEs is retained.

**Metric (gnomAD v.3.12)**	**cEEs (n = 870,139)**	**Ctrl- (n = 236,177)**	**Test (Two-sided)**
Median AF of all variants	6.6e-06	8.0e-06	Mann-Whitney p = 0
Ultra-rare fraction (AF ≤ 0.01 %)	90.9 %	90.4 %	χ^2 p = 1.8 × 10^{-13}
Median nb of synonymous SNVs normalized per exon size	0.065	0.042	Mann-Whitney p = 0
Median Synonymous/All variant ratio	0.329	0.265	Mann-Whitney p = 0
pN/pS<br>(gnomAD missense / synonymous)	All: 0.584<br>per-exon median: 0.587	All: 0.611<br>per-exon median: 0.551	χ^2 p = 1.3e-137<br>Mann-Whitney p = 1.09e-21
dN/dS<br>(human-mouse orthologues)	All: 0.427<br>per-exon median: 0.086<br>n = 7,802	All: 0.311<br>per-exon median: 0.088<br>n = 8,095	χ^2 p = 2.1e-13<br>Mann-Whitney p = 6.2e-4
Direction of Selection (DoS) median	0.042<br>n = 7,298	-0.083<br>n = 5,982	Mann-Whitney p = 4.2e-39
Fixation Index (*FI*) median	1.235<br>n = 7,298	0.666<br>n = 5,982	Mann-Whitney p = 4.7e-43

Table S3: Allele-frequency distribution of gnomAD/TCGA SNVs in cEEs versus control exons.

The upper panel reports global gnomAD[1] v3.1.2 allele frequencies; the lower panel repeats the analysis with TCGA[2] tumour-exome calls. Values are percentages of the total variant count in each set. A two-proportion *z*-test (two-sided) compares the cEE proportion with the control proportion for each frequency bin; NS, not significant at $p > 0.05$.

gnomAD statistics. Raw counts (cEEs 870 139; controls 236 177 variants) were first analysed with a 3 × 2 χ^2 test ($\chi^2 = 140.9$, d.f. = 2, $p = 2.5 \times 10^{-31}$). Collapsing to “rare” (AF ≤ 0.1 %) versus “non-rare” (AF > 0.1 %) gave an odds ratio of 1.15 (95 % CI 1.12–1.18; Fisher’s exact $p = 2.9 \times 10^{-28}$), indicating a modest but highly significant enrichment of very low-frequency variants within cEEs.

TCGA statistics. TCGA somatic SNVs are dominated by common alleles after gnomAD masking; no significant differences are observed between cEEs and controls (all $p > 0.05$).

Note: allele frequencies are calculated across all gnomAD populations; ancestry-specific frequencies were not considered.

**Allele-frequency bin (gnomAD v.3.12)**	**cEEs (n = 870,139)**	**Ctrl- (n = 236,177)**	**Two-Proportion Z-Test (Two-sided)**
Ultra-rare (AF <=0.01%)	90.93 %	90.44 %	1.82e-13
Rare (0.01% < AF <= 0.1%)	6.00 %	6.04 %	NS
Low-freq (0.1% < AF <= 1%)	1.87 %	2.06 %	9.51e-09
Common (AF > 1%)	1.18 %<br>100%	1.44 %<br>100%	2.94e-25<br>NS

**Allele-frequency bin (TCGA)**	**cEEs (n = 393,251)**	**Ctrl- (n = 131,647)**	**Two-Proportion Z-Test (Two-sided)**
Ultra-rare (AF <=0.01%)	0%	0%	NS
Rare (0.01% < AF <= 0.1%)	0%	0%	NS
Low-freq (0.1% < AF <= 1%)	0%	0%	NS
Common (AF > 1%)	100%	100%	NS

Table S4: cEEs tested by reporter luciferase assay Figure 3a.

List of candidate EE genomic regions tested by luciferase assay.

EE ID	Sequences
EE1	chr7:139600417-139600596
EE2	chr2:33020909-330212
EE3	chr19:34327990-34328151
EE4	chr2:85326508-85326804
EE5	chr20:3744902-3745088
EE6	chr1:212007250-212007449
EE7	chr7:105106522-105106772
EE8	chr10:28120194-28120390
EE9	chr12:103997451-103997535
EE10	chr10:27173118-27173259
EE11	chr14:88472244-88472465
EE12	chr11:121553937-121554109
EE13	chr1:29033093-29033245
EE14	chrX:71394067-71394245
EE15	chr11:74085618-74085886
EE16	chr1:113659080-113659265
EE17	chr19:50414815-50414990
EE18	chr11:27372283-27372398
EE19	chr9:129863186-129863299
EE20	chr3:179375220-179375341
EE21	chr6:34835294-34835464
EE22	chr15:41438321-41438431
EE23	chr6:29631396-29631599

Table S5: Tested promoters on Figure 3bc.

**GARRE1 Promoter (P1 GARRE1)**	chr19:34254350-34254697
**GPI Promoter (P2 GARRE1)**	chr19:34364882-34365266
**Promoter PDCD2L (P3 GARRE1)**	chr19:34404188-34404430
**Promoter USP20 (P1 USP20)**	chr9:129835222-129835554
**Promoter C9orf78 (P2 USP20)**	chr9:129835222-129835554
**Promoter TOR1B (P3 USP20)**	chr9:129802932-129803212

Table S6: cEEs mutated on Figure 3de.

Sequences of wt and mutated cEEs tested by luciferase

EE3_WT
GGCTGCTGCAGCGAGGCGGAAGCCCAGCAGACGGGGCGGAGGCAGACACCCCCGCAGCCCATGCAGT<br>GTGAGCTCCCCACCGTCCCTGTGCAGATAGGATCGCACTTCCTGAAGGGCGTCTCCTTTAATGAGTCGGC<br>CGCCGACAATCTGAAACTTAAGACG
EE3_gnomaD
GGCTGCTGCAGCGAGttaGAAGCCCAGCAGACGGGGaaGAGGCAGACAgCCtCaCAGCCCATGCAGTGTGA<br>GCTCCCCACtaTCCCTGTGCAGATAGGATtcCACTTtCTGAAGGGtGTCTtCTTgAATGAGTtGGCtaCCGACAgT<br>CTGAAACTTAAGAgG
EE3_synonymous
GGCTGCTGCAGCGAGGCaGAAGCCCAGCAGACGGGGaGGAGGCAGACACCCCCaCAGCCCATGCAGTG<br>TGAGCTCCCCACtGTCCCTGTGCAGATAGGATCcCACTTtCTGAAGGGtGTCTCCTTcAATGAGTCGGCtGCC<br>GACAATCTGAAACTTAAGACG
EE3_missense
GGCTGCTGCAGCGAGttGGAAGCCCAGCAGACGGGGCaGAGGCAGACAgCCtCGCAGCCCATGCAGTGTG<br>AGCTCCCCACCaTCCCTGTGCAGATAGGATtGCACTTCCTGAAGGGCGTCTtCTTgAATGAGTtGGCCaCCG<br>ACAgTCTGAAACTTAAGAgG
EE19_WT
CCCGCCGCTGACTCAGTTCTTCTTGGAGTGTGGCGGCCTGGTGCGCACAGATAAGAAGCCAGCCCTGTG<br>CAAGAGCTACCAGAAGCTGGTCTCTGAGGTCTGGCATAAGAAACG
EE19_ in silico
CCCGCCGCTcACcCAGTTCTTtTTGGAGTGTGGCGGCCTGGTGCGCACgGAcAAGAAGCCAGCCCTGTGC<br>AAGAGCTACCAaAAGCTGGTCTCTGAGGTCTGGCATAAGAAACG
EE19_gnomaD
CCCGCCGCTGACTtAaTTCTTCTTcGAGTGTGGCGGCCTGGTGCGCACAGggAAGAAGCCAGCCCTGTGC<br>AAGAGCTACCAGAAGCTGGTCTCTGAGGTCTGGCATAAGAAAtG
EE20_WT
GAGTGTATCTCGCAGTCAGCAGTGAAAACAAAGTTCGAACAGCACACTATCAGAGCTAAACAGATACTAGC<br>TACTGTGAAAAACATAATGGATTCAGTAAACCTGGCAGCTGAAGATAAAAG
EE20_in silico
GAGTGTATCTCaCAGTCAGCtGTGAAAACAAAGTTgGAACAGCACACTATCAGAGCTAAACAaATACTAGCTA<br>CTGTGAAAAACATAATtGATTCtGTAAACtTGGCAGCTGAAGATAAgAG

Table S7: gRNAs used for CRISPRi Figure 6b-e.

Name	Sequence
GARRE1_gRNA_CRISPRi	CCGACAATCTGAAACTTAAG
USP20_gRNA_CRISPRi	CCGCTGACTCAGTTCTTCTT
STK11IP_gRNA_CRISPRi	TCCGGAAACCCTCTGCCGGC
COG1_gRNA_CRISPRi	TTGACAGATACGCAGATGCG
gRNA no target	GGGAACGACTATGACCGCCA
CD81_gRNA_CRISPRi	GCCTGGCAGGATGCGCGGTG

Table S8: Primers to assess gene expression Figure 6b-e, Supp Figure S28.

Gene	Forward	Reverse
qGARRE1	CAGCAGTGGAGAGCAAGACAC	CTTTGGGTGGCCACGTTTTG
qGARRE1_up ex6	AAAAATCGACAGTGCTTTGC	CTTTGGATTCAGGTGGAAGC
qUSP20	TCAAAGGAAGCGGCCATGTC	CAGGCTGTTCAGGTAGGTCC
qUSP20_up ex9	CTGAAAGCTGTTCCTATTGC	CAGCGTTCATGTAGCAGGAG
qSTK11IP	CGGCTGCTCTTCTACGATG	AACAGCTCCTCCCATGGTTC
qSTK11IP_up ex14	AGCCCCTGCTTCATAAGGTT	GGAGAGGGGTTCAGAGTTCG
qCOG1	ACATCGAAACAAAAGCTCAGGT	GTCTTCTTCACTGACAAGCTGT
qSUCO	ACCCCTCAAGTTTTCTCCAG	TGGGGTGCAATGGTTCTTC
qPKD1L2	ATCAGCTGTCGGAGGAAGGG	CAGAGACCCAGGCTGCTC
qGPI	TCGTTCAGGGCATCATCTGG	TCACTTGAGCACTGCCATC
qC9orf78	GGCCCTTGAGAGTAGGTGAC	TGCCTTCTCGTTAGCAGGAC
qOBSL1	GTTCGACCTGAGGACCAAGG	CCTAGTTGCCCTCTACCAGC
qVCF1	CAGACTAAGAGGAAACAAAGCGT	CTGTCCGGGCTATTGATGCT
qPIGC	CCATTCTACCTCATTCGCTTGC	ACTGAGGAACCTGGACAAGTC
qGCSH	CTCTTGCAGAAAATCCAGGACT	AGGGTTACTCAGTGTCATCTTG
qPDCD2L	GCTCAAGAGTGCTAATTTAGGTC	GGGGCCAGCAACTCTTCTC
qTOR1B	CAGCTGAAGGACCTGGAACC	AGGCAGGAAGGGGATAAAGT
qCHPF	CCTGTGCACATGTACCAGCT	TCCCACTGTAACTCCTGGATCT
qC17orf80	CGTCCTGTGTTGTAGCTGGA	TCTCCACTGTACGCCCTTTG
qCD81	TTCTCCGGGAAGCTGTACCT	ACCATGCTCAGGATCATCTCG

Supplementary figures

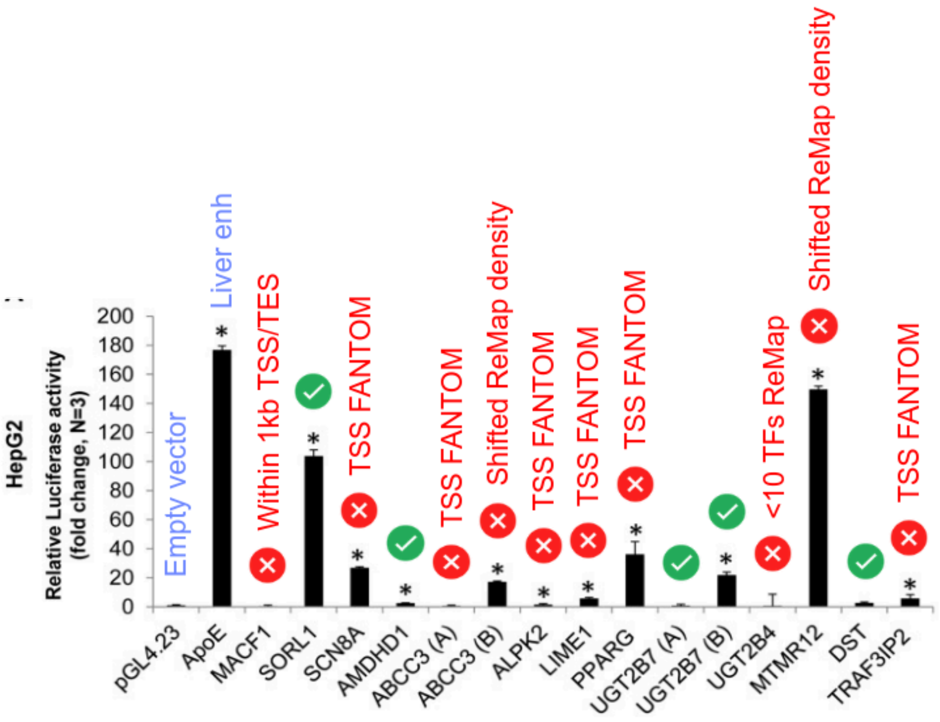

Figure S1: Re-evaluation of exon enhancer candidates from Birnbaum *et al*.

Barplot representing a re-evaluation of enhancer activity for 15 exon candidates originally tested in HepG2 cells, reported by Birnbaum *et al*.[3]. Exons were lifted to hg38 assembly and filtered based on our stringent exon enhancer selection criteria. Excluded exons are labelled in red along with the exclusion parameters, including proximity to transcription start site (TSS) or transcription end site (TES), overlap with FANTOM[4,5] TSS, shifted ReMap[6] ChIP-seq peaks density, or insufficient TF ChIP-seq peaks (<10 TFs in ReMap). The y-axis represents relative luciferase activity in HepG2 cells (fold change, n ≥ 3). Black bars indicate enhancer activity levels, with significant changes marked by asterisks. The control empty vector and a known liver enhancer are included as references.

This figure is adapted from Figure 1A from Birnbaum *et al.*[3], which is published under a Creative Commons Attribution (CC-BY) licence; reuse and adaptation are permitted with attribution. Experimental design and statistical analyses are described in the original publication (Birnbaum RY, PLOS Genet. 2014), whereas exon filtering and annotations were re-evaluated in this study.

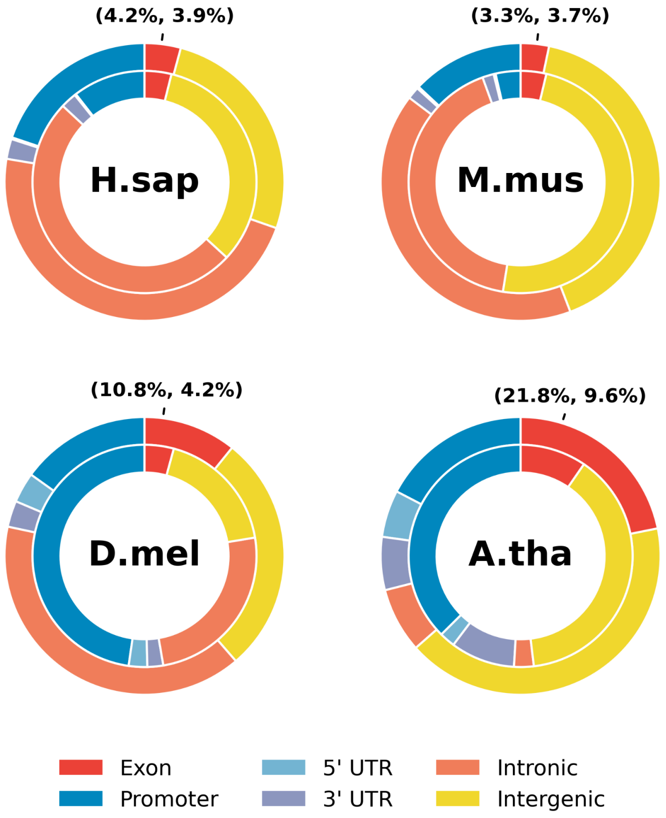

Figure S2: Genomic distribution of ChIP-seq and DNase-seq data across four species.

The outer layer shows the genomic distribution of non-redundant transcription factor ChIP-seq summits from ReMap2022[6] across *Homo sapiens* (*H.sap*), *Mus musculus* (*M.mus*), *Drosophila melanogaster* (*D.mel*), and *Arabidopsis thaliana* (*A.tha*), annotated using ChIPseeker v3.20. The inner layer shows the distribution of DNase-seq sites obtained from Meuleman *et al.* for *H. sapiens*, ENCODE for *M. musculus*, ChIP-Atlas for *D. melanogaster*, and PlantRegMap for *A. thaliana*. Genomic categories include promoter, exon, intron, 5′ UTR, 3′ UTR, and intergenic regions.

Percentages shown above each species indicate, respectively, the proportion of TF ChIP-seq summits (outer ring) and DNase-seq sites (inner ring) located in exonic regions. For example, in *M. musculus*, 3.3% of ReMap ChIP-seq summits and 3.7% of DNase-seq sites overlap coding exons.

Non-redundant transcription factor ChIP-seq peak counts from ReMap2022 were ~68.2 million for *Homo sapiens* (hg38), ~43.9 million for *Mus musculus* (mm39), ~12.9 million for *Drosophila melanogaster* (dm6), and ~3.2 million for *Arabidopsis thaliana* (TAIR10) (ReMap2022 catalog). DNase-seq dataset counts per species were n = 1,744,401 (*Homo sapiens*), n = 1,639,428 (*Mus musculus*), n = 714,638 (*Drosophila melanogaster*), and n = 806,019 (*Arabidopsis thaliana*).

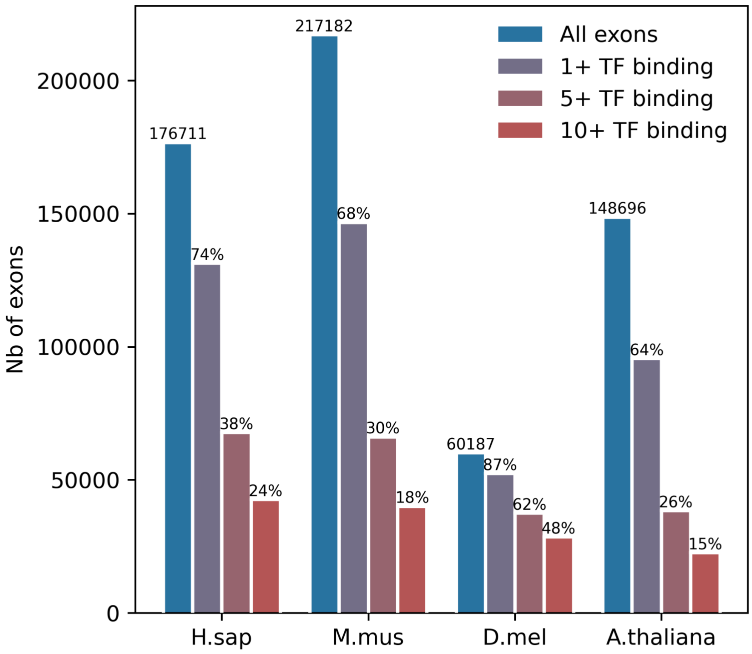

Figure S3: Transcription factor occupancy in coding exons across species.

Bar plot showing the number of merged protein-coding exons overlapping non-redundant transcription factor (TF) ChIP-seq summits from ReMap2022 across four species: *Homo sapiens* (*H.sap*), *Mus musculus* (*M.mus*), *Drosophila melanogaster* (*D.mel*), and *Arabidopsis thaliana* (*A.tha*). Bars indicate the total number of exons (blue) and subsets with different degrees of TF binding: ≥1 TF (grey), ≥5 TFs (brown), and ≥10 TFs (red). Percentages above each bar represent the proportion of exons in each binding category relative to the total number of exons for that species. Total sample sizes for all coding exons (n) are indicated directly above the blue bars for each species.

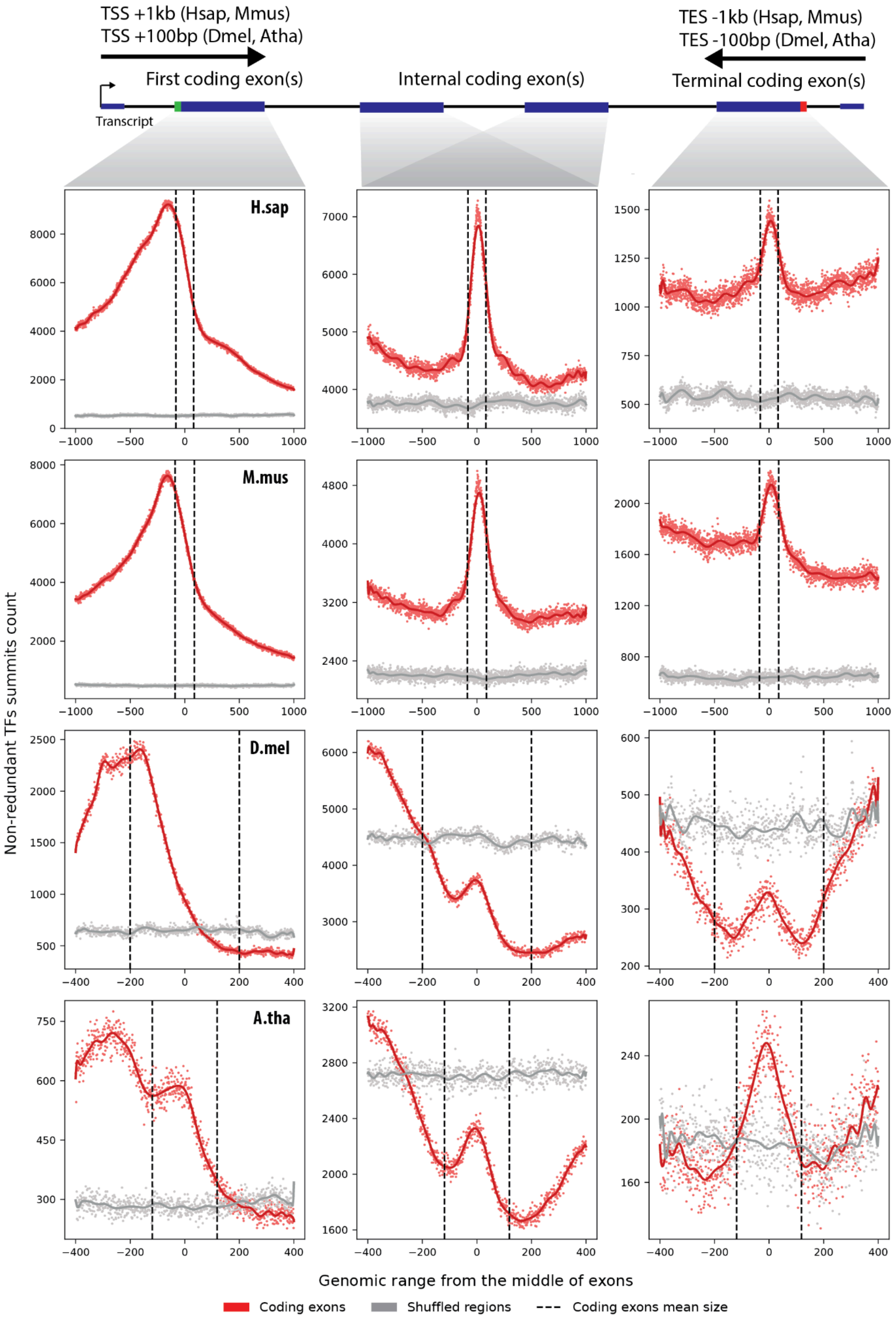

Figure S4: Genomic distribution of transcription factor binding across exon classes.

Meta-profile analysis showing genomic distribution of non-redundant transcription factor (TF) ChIP-seq summits from ReMap2022 over merged protein-coding exons across four species: *Homo sapiens* (*H.sap*), *Mus musculus* (*M.mus*), *Drosophila melanogaster* (*D.mel*), and *Arabidopsis thaliana* (*A.tha*). The top schematic categorizes exons into three regions: transcription start site (TSS)-proximal exons (±1000 bp for *H.sap* and *M.mus*; ±100 bp for *D.mel* and *A.tha*), internal coding exons, and transcription end site (TES)-proximal exons (±1000 bp for *H.sap* and *M.mus*; ±100 bp for *D.mel* and *A.tha*). The line plots represent the distribution of TF ChIP-seq summits across exonic regions in each category and each species. TF occupancy in coding exons is shown in red, while shuffled control regions are represented in grey. Dashed vertical lines indicate mean exon boundaries. This analysis reveals TF-binding enrichment patterns along exons.

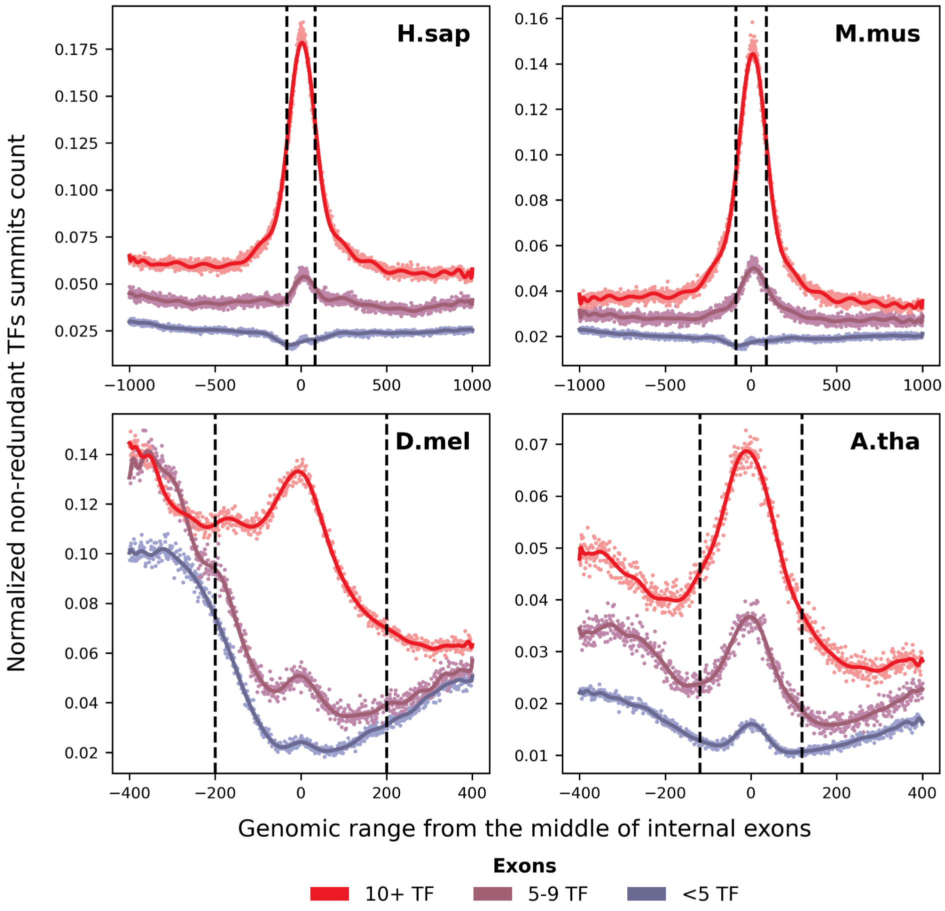

Figure S5: Transcription factor binding density in internal exons.

Meta-profile analysis showing the normalized distribution of non-redundant transcription factor (TF) ChIP-seq summits from ReMap2022 in internal exons of *Homo sapiens* (*H.sap*), *Mus musculus* (*M.mus*), *Drosophila melanogaster* (*D.mel*), and *Arabidopsis thaliana* (*A.tha*). Data are stratified by TF binding density: exons bound by <5 TFs (blue), 5-9 TFs (purple), and ≥10 TFs (red). The x-axis represents the genomic distance (bp) from the center of internal exons, with dashed vertical lines indicating average exon boundaries for each species. The y-axis represents the normalized frequency of TF ChIP-seq summits. Lines show the smoothed average profile, while dots represent individual data points. The data reveal a clustering of TF binding around exon centers, with a stronger enrichment in exons containing a higher number of TF peaks, highlighting potential. Sample sizes were: *Homo sapiens* (hg38) cEEs, n = 13,481; *Mus musculus* (mm39) cEEs, n = 12,244; *Drosophila melanogaster* (dm6) cEEs, n = 13,688; and *Arabidopsis thaliana* (tair10) cEEs, n = 7,138.

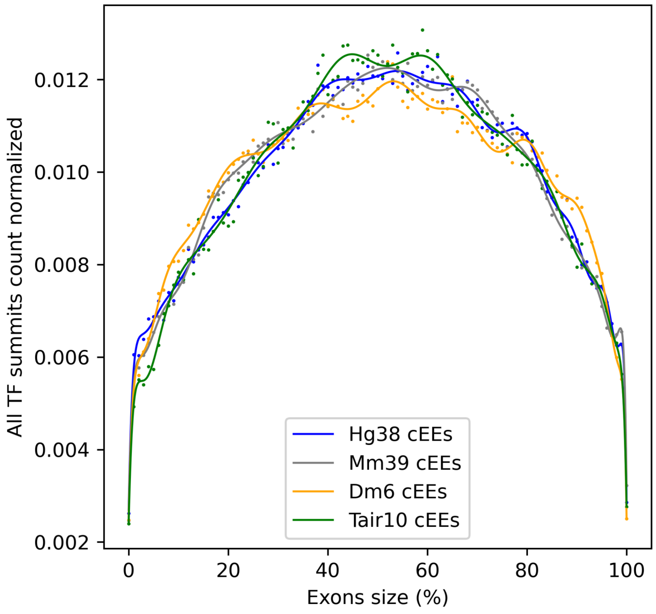

Figure S6: Position of TF-binding summits along cEEs in four species.

Smoothed density of non-redundant ReMap2022 TF-ChIP summits plotted along the length of cEEs in *H. sapiens* (blue), *M. musculus* (grey), *D. melanogaster* (orange) and *A. thaliana* (green). Each exon was scaled to a 0–100 % coordinate system anchored at the 5′ and 3′ splice sites; dots show per-bin means, solid lines show LOESS fits. All four species display a pronounced reduction in summit density near splice junctions and a maximum in the internal half of the exon, confirming that the enrichment observed in main Fig. 1E is not an artefact of midpoint centring but reflects a conserved positional bias of TF binding within coding exons. Sample sizes were: *Homo sapiens* (hg38) cEEs, n = 13,481; *Mus musculus* (mm39) cEEs, n = 12,244; *Drosophila melanogaster* (dm6) cEEs, n = 13,688; and *Arabidopsis thaliana* (tair10) cEEs, n = 7,138.

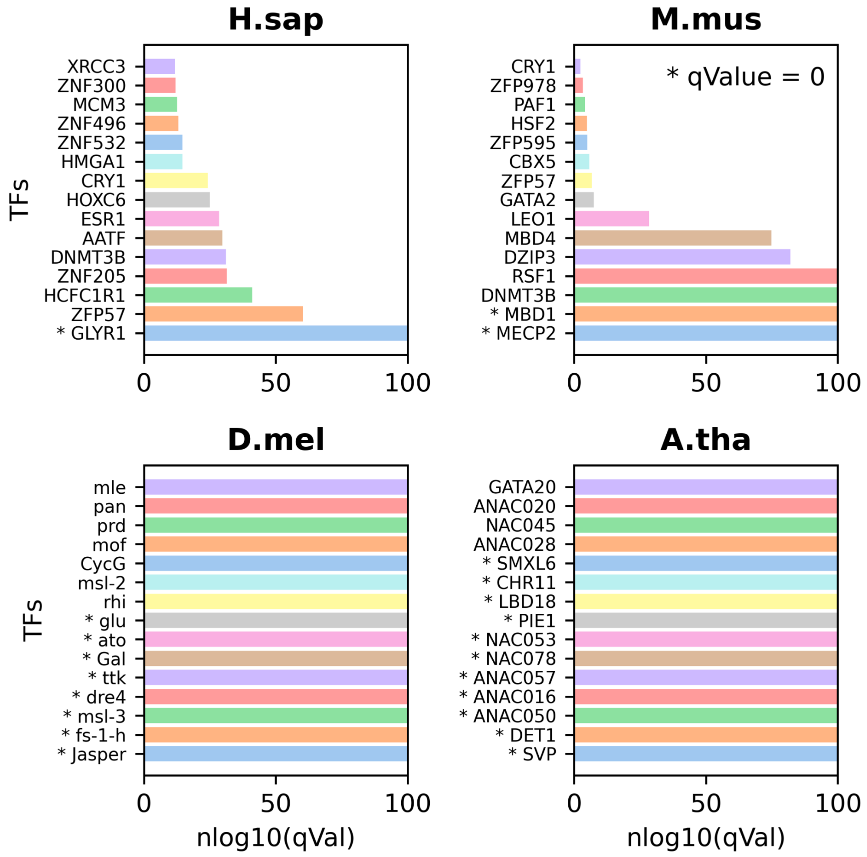

Figure S7: Enrichment of transcription factors in cEEs.

Enrichment analysis of transcription factors (TFs) binding to candidate Exonic Enhancers (cEEs) was performed using non-redundant ReMap2022 ChIP-seq datasets across four species: *Homo sapiens* (*H.sap*), *Mus musculus* (*M.mus*), *Drosophila melanogaster* (*D.mel*), and *Arabidopsis thaliana* (*A.tha*). Enrichment was assessed using LOLA[7] (v.1.12.0) by comparing cEE-associated TFs to promoter-associated TFs from the Eukaryotic Promoter Database[8] (EPD, https://epd.expasy.org/epd/).

The top 15 enriched TFs in each species are ranked by -log10(q-value; computed by LOLA with a Fisher's exact test followed by a FDR correction), with significantly enriched TFs marked with an asterisk. *H.sap* and *M.mus* show strong enrichment for ZNF-family proteins, chromatin remodelers, and DNA methylation regulators, while D.mel and A.tha exhibit enrichment for species-specific transcription factors involved in developmental regulation. These results highlight the distinct TF repertoires associated with cEEs across species.

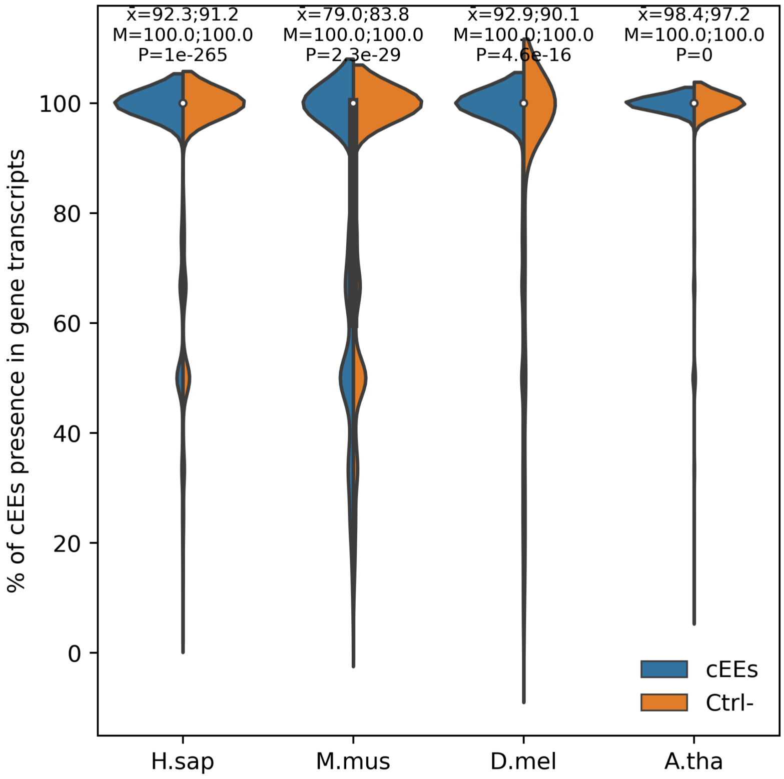

Figure S8: Prevalence of cEEs across gene transcript isoforms.

For every protein-coding gene we calculated the percentage of its gene transcripts that contain a given cEE and compared the resulting distributions (violin plots) with negative control exons (Ctrl–). Results are shown for *Homo sapiens* (*H.sap*), *Mus musculus* (*M.mus*), *Drosophila melanogaster* (*D.mel*) and *Arabidopsis thaliana* (*A.tha*). Numbers above each violin give the mean (x̄) and median (M) proportion for cEEs and controls, respectively; p values computed from Two One-Sided Tests (TOST) procedure with unequal variances to test the equivalence between the two distributions.

In all four species, the median is 100 % for both sets, but the mean proportion is consistently higher for cEEs, indicating that most cEEs are present in nearly every transcript isoform of their host gene. This widespread inclusion suggests that cEEs are generally retained during alternative splicing and may therefore influence gene regulation across the full transcript repertoire.

Violin plots show the distribution of values, with the width of each violin proportional to the data density; the central point indicates the median, and the vertical extent represents the full range of the data. Sample sizes were: *H. sapiens* cEEs (n = 13,481) and Ctrl− exons (n = 13,253); *M. musculus* cEEs (n = 12,244) and Ctrl− exons (n = 18,457); *D. melanogaster* cEEs (n = 13,688) and Ctrl− exons (n = 903); *A. thaliana* cEEs (n = 7,138) and Ctrl− exons (n = 7,862).

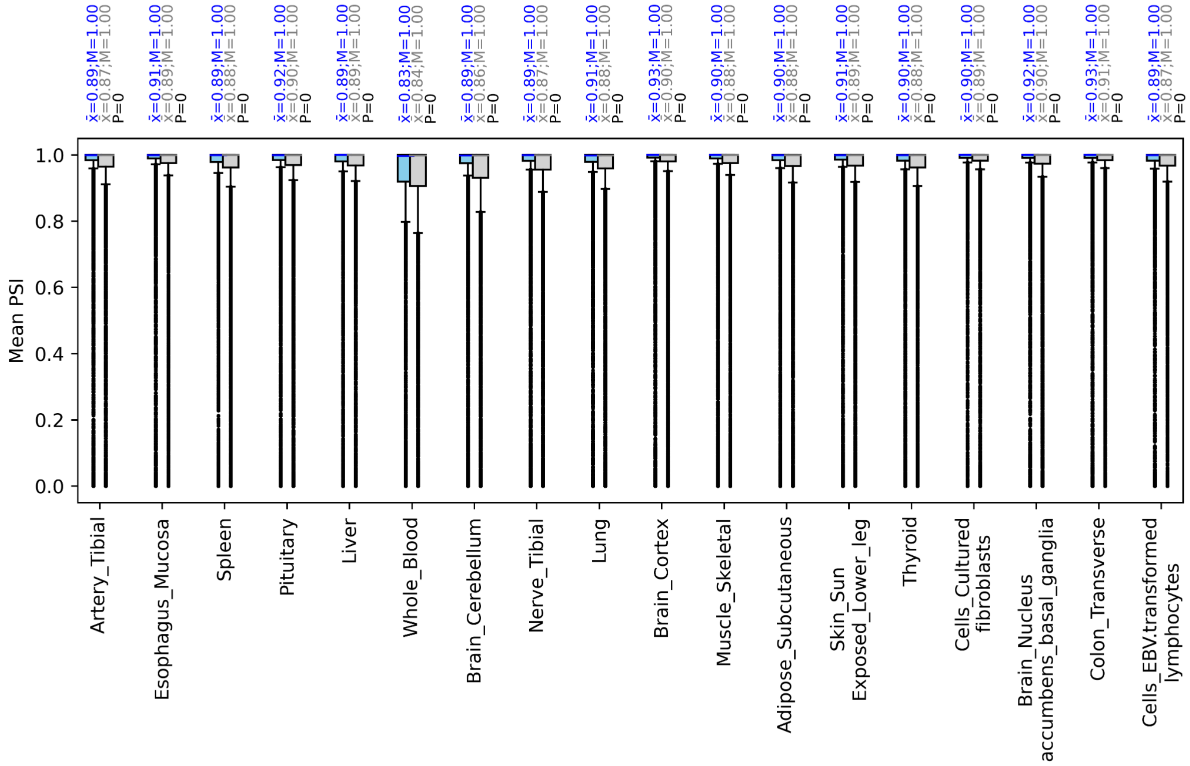

Figure S9: Constitutive inclusion of cEEs across GTEx tissues.

For each of 18 GTEx tissues, box-and-whisker plots show the distribution of mean percent-spliced-in (PSI) values for every candidate exonic enhancer (cEE, blue) and for length/GC-matched control exons (grey). For each exon, PSI was averaged across all samples in the tissue; NA values were excluded. Numbers above each pair of boxes give, left-to-right, the tissue-specific mean ($\bar{x}$) and median (M) PSI for cEEs, followed by the corresponding values for controls, and the p values computed from Two One-Sided Tests (TOST) procedure with unequal variances to test the equivalence between for the cEE-vs-control comparison. In every tissue the median PSI equals 1.00 for both sets, indicating that the vast majority of cEEs and controls are constitutively included. The consistently higher mean PSI for cEEs and the uniformly significant p values (all $p < 10^{-16}$, reported as 0) reflect a slight but systematic shift toward more complete inclusion of cEEs, consistent with broad usage of cEEs across transcripts. Box plots show the median (centre line) and interquartile range (box, 25th to 75th percentiles); whiskers extend to the most extreme values within 1.5×IQR, with points beyond the whiskers plotted as outliers. Across tissues, analyses included 13,481 cEEs and 13,253 matched control exons.

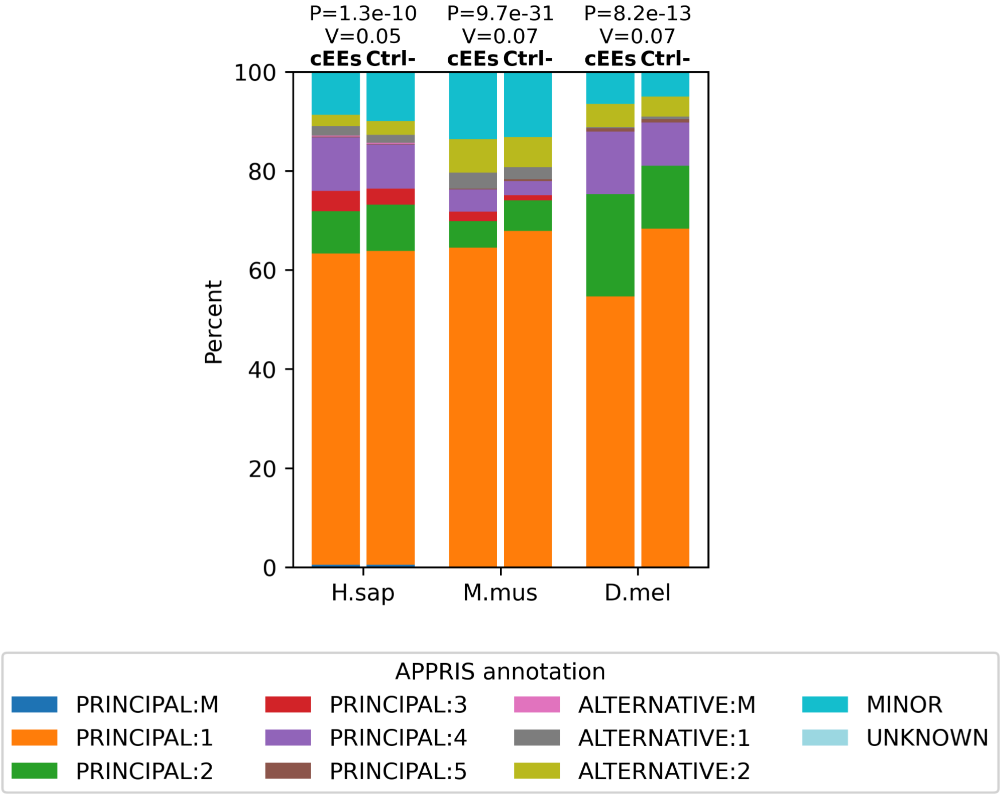

Figure S10: APPRIS isoform annotation of cEEs versus control exons.

Stacked bars show the distribution of APPRIS[9] v50 categories for cEEs (left bar of each pair) and negative-control exons (Ctrl–) (right bar) in H. sap, M. mus, and D. mel. All APPRIS labels are displayed (PRINCIPAL:M/1/2/3/4/5; ALTERNATIVE:M/1/2; MINOR; UNKNOWN); percentages sum to 100% per bar.

For statistical comparison, APPRIS categories were collapsed into three classes: principal (PM/P1/P2), other principal (P3–P5), and non-principal (ALTERNATIVE:M/1/2 + MINOR + UNKNOWN). Differences between cEEs and Ctrl− exons were assessed using a χ^2 test (two-sided); χ^2 p values and Cramér's V are shown above each species pair (*H. sapiens*: $p = 1.3 \times 10^{-10}$, $V = 0.05$; *M. musculus*: $p = 9.7 \times 10^{-31}$, $V = 0.07$; *D. melanogaster*: $p = 8.2 \times 10^{-13}$, $V = 0.07$), indicating statistically significant but small effect sizes consistent with broadly similar APPRIS profiles between cEEs and control exons.

Sample sizes were as follows: for *Homo sapiens*, cEEs (n = 13,481) and Ctrl− exons (n = 13,253); for *Mus musculus*, cEEs (n = 12,244) and Ctrl− exons (n = 18,457); and for *Drosophila melanogaster*, cEEs (n = 13,688) and Ctrl− exons (n = 903).

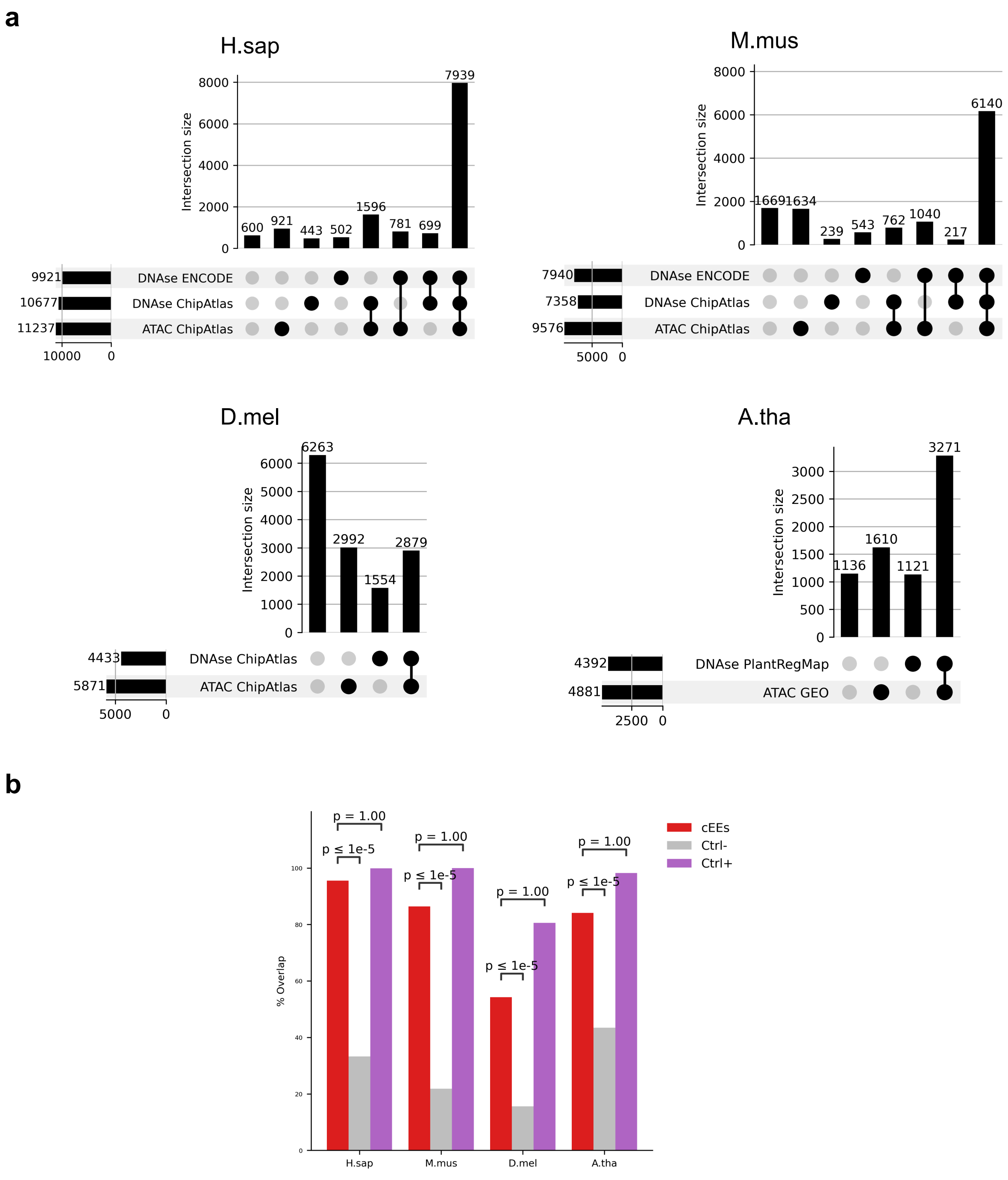

Figure S11: Chromatin accessibility at exon enhancers across species.

(a) Overlap of candidate exon enhancers (cEEs) across four species with DNase-seq and ATAC-seq peaks from ENCODE[10], ChIP-Atlas[11], PlantRegMap[12], and GEO datasets. UpSet plots depict the intersection sizes between cEEs and chromatin accessibility datasets, highlighting the fraction of cEEs supported by one or multiple independent sources. **(b)** Quantification of chromatin accessibility overlap for cEEs (n = 13,481), control exons lacking TF binding (Ctrl-, n = 13,253), and highly bound control intergenic enhancers (Ctrl+, n = 404,325) across species (*M. musculus* Ctrl- n = 18,457, cEEs n = 12,244, Ctrl+ n = 149,241 ; *D. melanogaster* Ctrl- n = 903, cEEs n = 13,688, Ctrl+ n = 133,253 ; *A. thaliana* Ctrl- n = 7,862, cEEs n = 7,138 Ctrl+ n = 9,025). Differences in the proportion of elements overlapping DNase-seq or ATAC-seq peaks were assessed using Fisher's exact one-sided test. P-values are indicated on the figure.

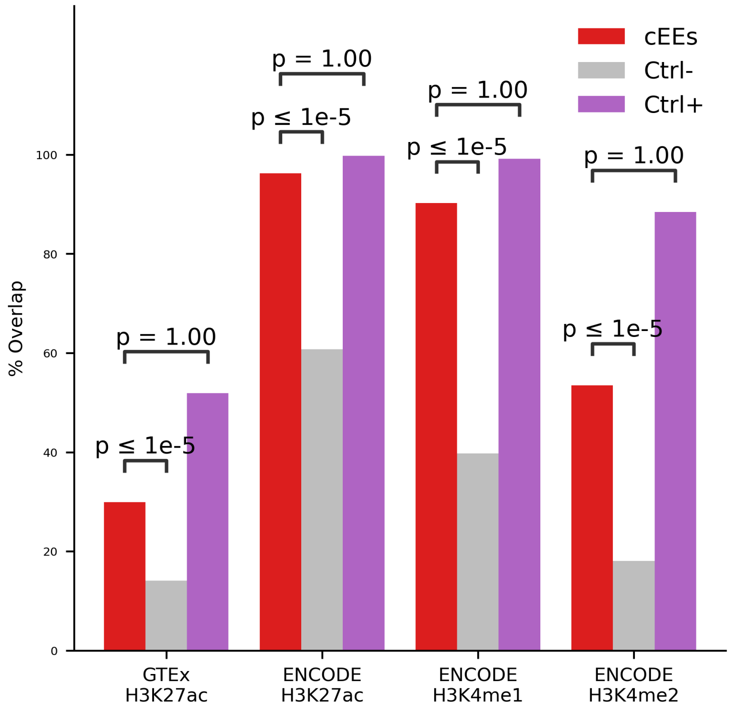

Figure S12: Enrichment of histone modifications at exon enhancers.

Histone modification enrichment at candidate exon enhancers (cEEs) in *Homo sapiens*. Overlap of cEEs (n = 13,481) with H3K27ac marks from GTEx (5 tissues) and ENCODE (853 datasets), as well as with H3K4me1 (388 datasets) and H3K4me2 (77 datasets) from ENCODE. The proportion of cEEs overlapping each histone modification was compared with negative control coding exons lacking TF binding (Ctrl-, n = 13,253) and highly bound control intergenic enhancers (Ctrl+, n = 404,325). Statistical significance of differences in overlap between cEEs and control sets was assessed using Fisher's exact one-sided test; p-values are indicated on the plot.

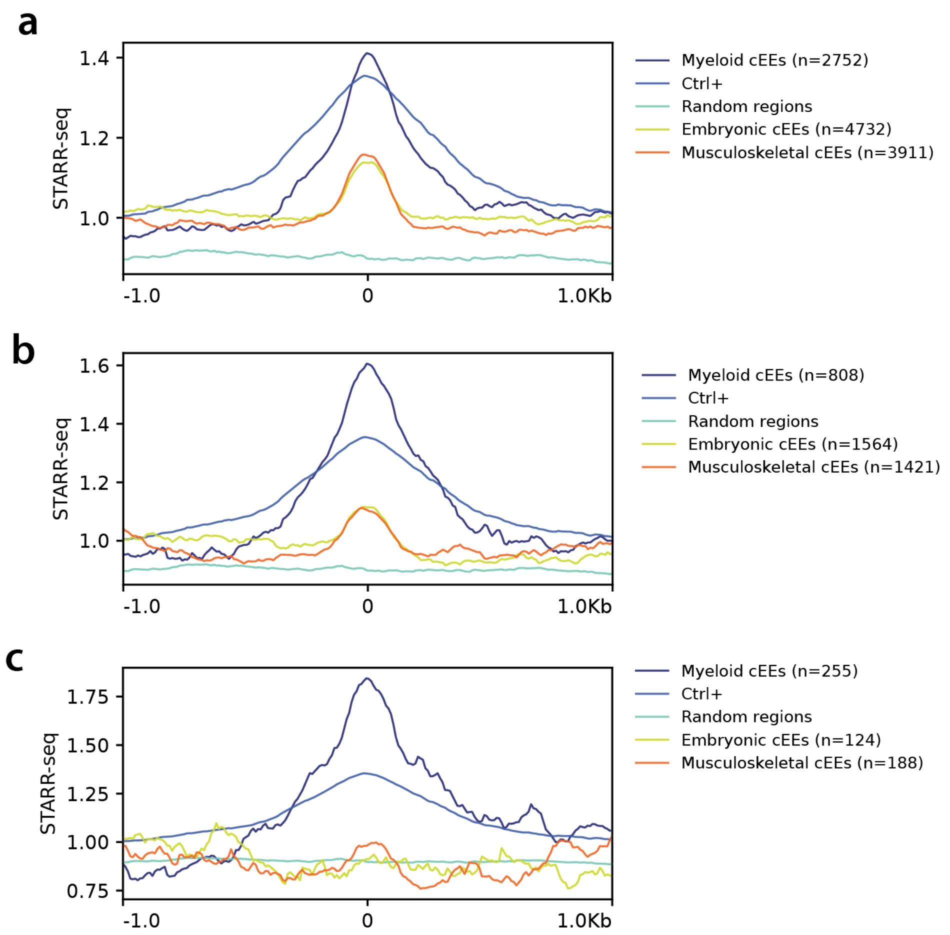

Figure S13: ENCODE STARR-seq activity in exonic enhancers stratified by biotype.

ENCODE STARR-seq signal intensity for identified candidate Exonic Enhancers (cEEs) in K-562 cells, categorized based on biotype specificity. **(a)** cEEs where myeloid TF ChIP-seq peaks are among the top three most frequent biotypes. **(b)** cEEs where myeloid TF ChIP-seq peaks represent the predominant biotype. **(c)** cEEs where myeloid TF ChIP-seq peaks are the predominant biotype, with at least 50% of cEE-associated transcription factors (TFs) linked to that biotype. Lines represent the mean STARR-seq signal across regions centered on the cEE midpoint; sample sizes are indicated on the figure. STARR-seq activity increases with myeloid-specific TF occupancy, with the strongest signal observed in cEEs enriched for myeloid TF ChIP-seq peaks. In contrast, STARR-seq activity progressively decreases in cEEs associated with embryonic and musculoskeletal biotypes, indicating that enhancer activity is shaped by cell-type-specific transcription factor binding.

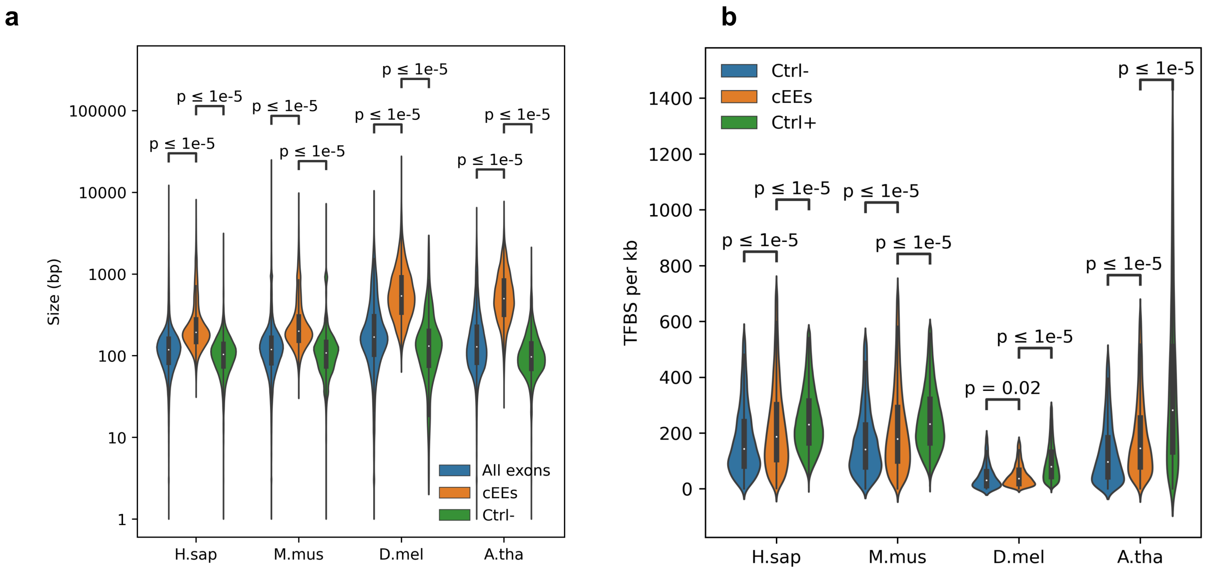

Figure S14: cEEs size and transcription factor binding site density.

(a) Size distribution of candidate exon enhancers (cEEs), control exons with no TF binding (Ctrl-), and all coding exons (excluding cEEs), across four species. cEEs appear larger than other coding exons. Sample sizes correspond to the full exon sets analysed per species (*H. sapiens*: All exons n = 163,230, cEEs n = 13,481, Ctrl- n = 13,253; *M. musculus* All exons n = 204,938, cEEs n = 12,244, Ctrl- n = 18,457; *D. melanogaster* All exons n = 46,499, cEEs n = 13,688, Ctrl- n = 903; *A. thaliana* All exons n = 141,558, cEEs n = 7,138, Ctrl- n = 7,862)
(b) Transcription factor binding site (TFBS) density per kilobase in cEEs across four species. TFBSs were predicted using JASPAR[13] 2024 (score ≥ 400) and intersected with cEEs, Ctrl- exons, and highly bound control exons (Ctrl+). Sample sizes correspond to (*H. sapiens*: Ctrl- n = 13,253, cEEs n = 13,481, Ctrl+ n = 404,325; *M. musculus* Ctrl- n = 18,457, cEEs n = 12,244, Ctrl+ n = 149,241 ; *D. melanogaster* Ctrl- n = 903, cEEs n = 13,688, Ctrl+ n = 133,253 ; *A. thaliana* Ctrl- n = 7,862, cEEs n = 7,138 Ctrl+ n = 9,025). Outliers were removed within each species and dataset using the interquartile range (IQR) method (values outside Q1 − 1.5×IQR or Q3 + 1.5×IQR).

Statistical significance was assessed using two-sided Student's t-tests with Bonferroni correction. Violin plots show the distribution of values, with width proportional to data density; central points indicate the median, and the vertical extent represents the range of observed values.

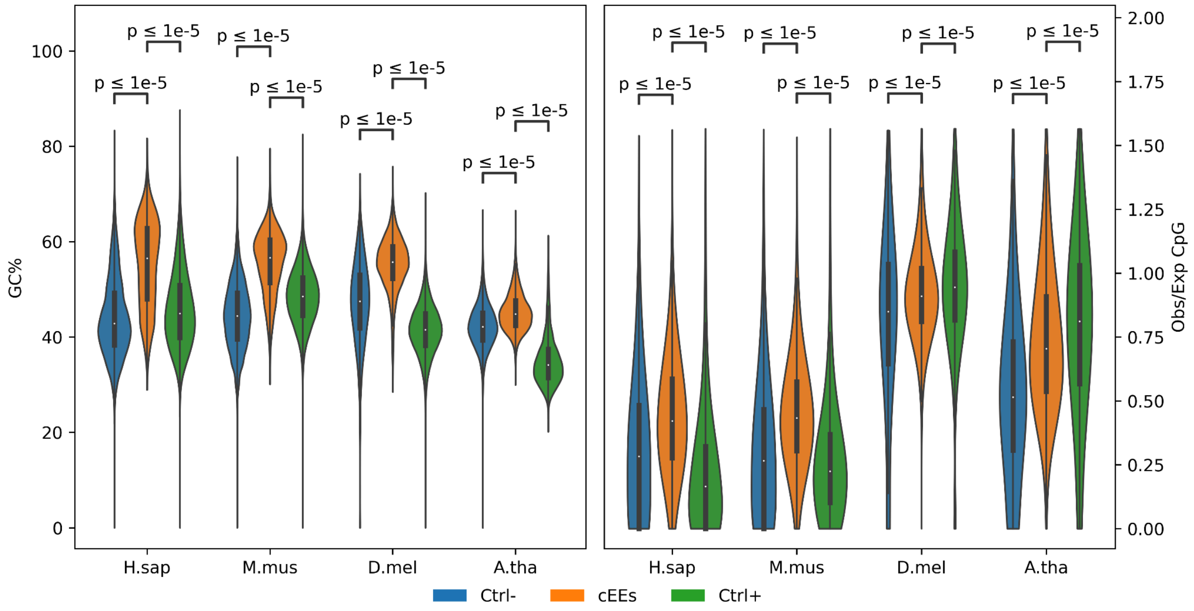

Figure S15: GC content and CpG enrichment at exon enhancers.

Comparative analysis of GC content and CpG observed-to-expected ratios at candidate exon enhancers (cEEs) across four species. The **left** panel shows GC content of cEEs compared with negative-control exons lacking TF binding (Ctrl-) and highly bound control exons (Ctrl+). The **right** panel shows the CpG observed-to-expected ratio ((CG)/(CxG)/L) for the same exon sets, with outliers removed within each species and dataset using the interquartile range (IQR) method (values outside Q1 − 1.5×IQR or Q3 + 1.5×IQR). Sample sizes correspond to the full exon sets analysed per species (*H. sapiens*: cEEs n = 13,481, Ctrl- n = 13,253, Ctrl+ n = 404,325; corresponding species-specific counts are provided in the Methods). Statistical significance was assessed using two-sided Student's t-tests with Bonferroni correction. Violin plots show the distribution of values, with width proportional to data density; central points indicate the median, and the vertical extent represents the range of observed values.

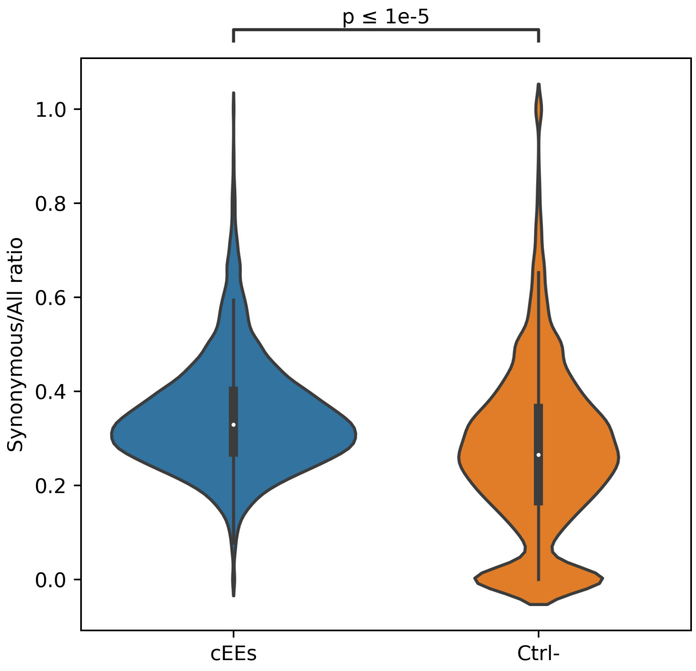

Figure S16: Synonymous-to-total variant ratio for cEEs versus matched coding exons.

Violin plots depict the distribution of the proportion of synonymous single-nucleotide variants (SNVs) among all gnomAD v3.1.2 SNVs per exon. Candidate exonic enhancers (cEEs; blue; n = 13,481) are compared with matched negative-control coding exons (Ctrl-; orange; n = 13,253). Horizontal bars mark the median values (0.33 for cEEs, 0.27 for controls; see Supplementary Table S2). Each data point corresponds to one exon. A two-sided Mann–Whitney test indicates that the difference is highly significant ($p < 10^{-300}$). Because synonymous substitutions are largely neutral at the protein level, the elevated synonymous-to-total ratio in cEEs implies a relative depletion of protein-altering variants, providing an additional population-genetic signature of purifying selection acting on cEE sequence. Violin plots show the distribution of values, with width proportional to data density; central points indicate the median, and the vertical extent represents the full range of observed values.

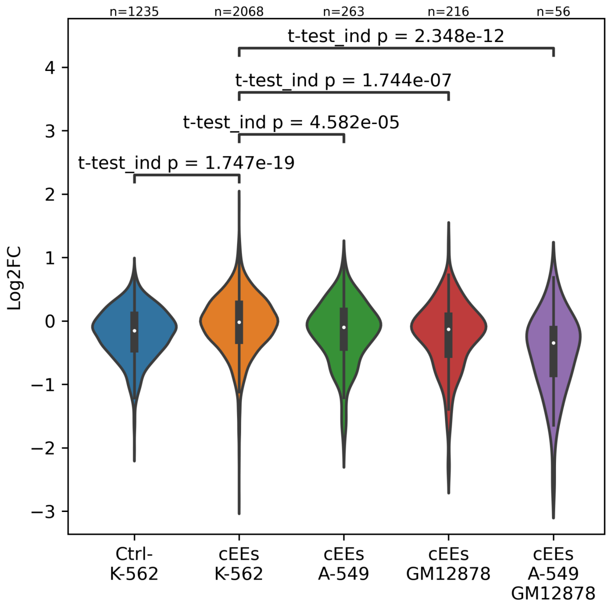

Figure S17: Cell line specificity and selection refinement of exon enhancer activity in STARR-seq.

The identified candidate Exon Enhancers (cEEs) exhibit significant differential activity across cell lines and selection criteria in STARR-seq assays. cEEs selected based on A-549 or GM12878 cell line signatures show significantly lower activity in K-562 cells, indicating context-dependent regulatory function. Sample sizes are indicated above each group (n = 1235, 2068, 263, 216, 56). Each data point represents one STARR-seq tested cEE. Statistical significance was assessed using two-sided Student's t-tests with Bonferroni correction for the indicated pairwise comparisons. Violin plots show the distribution of values, with width proportional to data density; central points indicate the median, and the vertical extent represents the full range of observed values.

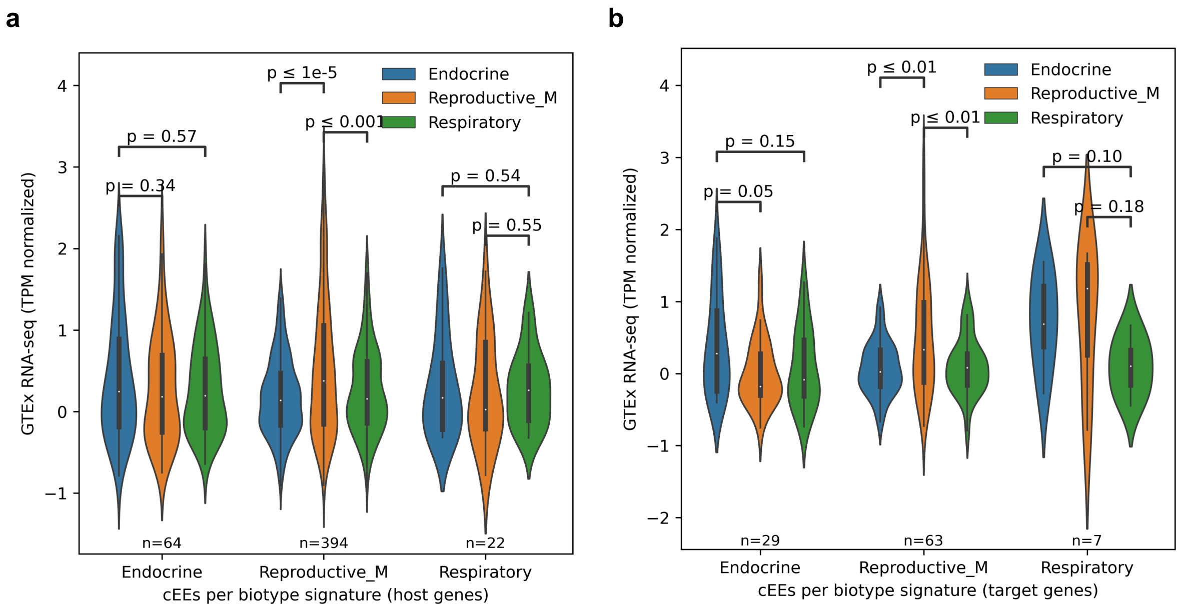

Figure S18: GTEx expression of cEE-associated genes.

(a) Violin plots showing normalised GTEx TPM values for host genes of cEEs carrying three exemplar biotype signatures (Endocrine, Reproductive_M, Respiratory). For each signature, expression in the matching tissue is compared with two randomly selected, non-matching tissues. *n* indicates the number of cEEs in each group. Outliers were removed within each species and dataset using the interquartile range (IQR) method (values outside Q1 − 1.5×IQR or Q3 + 1.5×IQR). Statistical significance was assessed using two-sided Mann–Whitney tests.

(b) Equivalent analysis for the predicted target genes of the same cEEs. Together, the panels illustrate that some biotype signatures are sharply enriched in their cognate tissue, whereas others display broad multi-tissue expression.

In all panels, violin plots show the distribution of values, with width proportional to data density; central points indicate the median, and the vertical extent represents the full range of observed values. These patterns mirror the mixture of tissue-restricted and broadly active profiles observed for canonical enhancers.

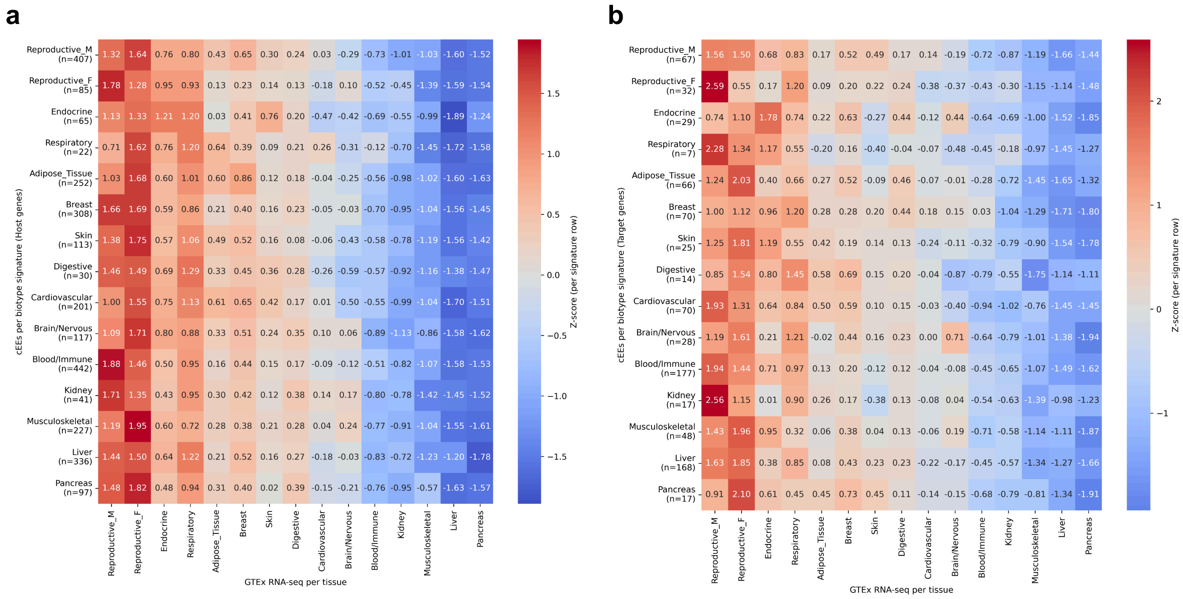

Figure S19: GTEx heatmap of cEE biotype groups.

Median normalised TPM values of cEE host genes **(a)** and their targeted genes across 18 GTEx tissues **(b)**. Each row represents one biotype group of cEE-associated genes. Expression values were converted to Z-scores within each biotype group, and rows are ordered by the rank and magnitude of the peak Z-score in the matching tissue. Colour intensity reflects relative expression levels, with warmer colours indicating higher Z-scored expression. The heatmaps show that, although tissue-matched enrichment is evident for some biotype groups, most groups retain appreciable expression across additional tissues. This pattern indicates that cEEs span a continuum from tissue-restricted to broadly active regulatory elements.

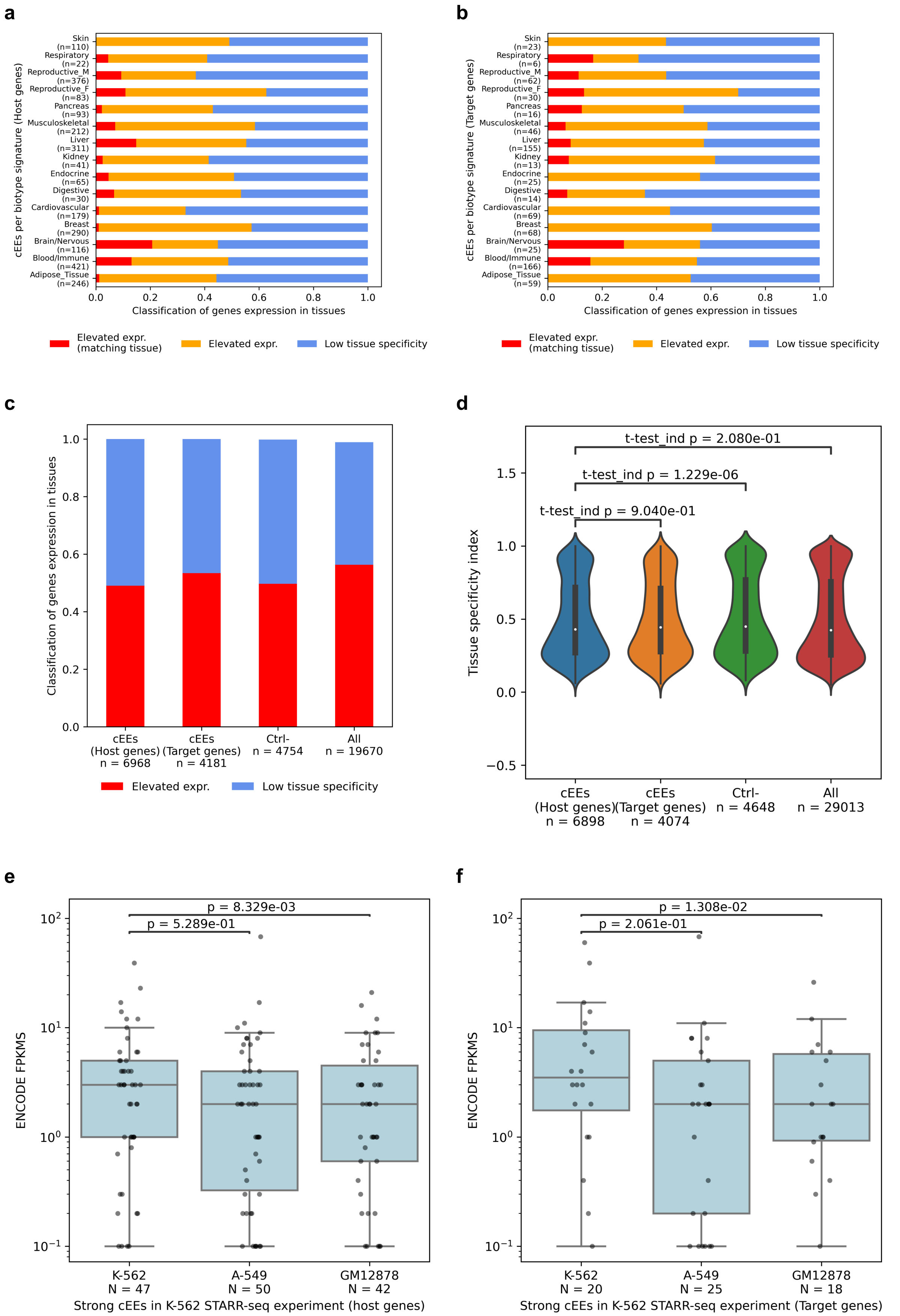
a
b
cEEs per biotype signature (Host genes)
cEEs per biotype signature (Target genes)
Classification of genes expression in tissues
Elevated expr. (matching tissue)
Elevated expr.
Low tissue specificity
c
Classification of genes expression in tissues
cEEs (Host genes) n = 6968
cEEs (Target genes) n = 4181
Ctrl- n = 4754
All n = 19670
d
t-test_ind p = 2.080e-01
t-test_ind p = 1.229e-06
t-test_ind p = 9.040e-01
Tissue specificity index
cEEs (Host genes) n = 6898
cEEs (Target genes) n = 4074
Ctrl- n = 4648
All n = 29013
e
p = 8.329e-03
p = 5.289e-01
ENCODE FPKMS
K-562 N = 47
A-549 N = 50
GM12878 N = 42
Strong cEEs in K-562 STARR-seq experiment (host genes)
f
p = 1.308e-02
p = 2.061e-01
K-562 N = 20
A-549 N = 25
GM12878 N = 18
Strong cEEs in K-562 STARR-seq experiment (Target genes)

Figure S20: Independent tissue-specificity metrics (HPA/Uhlén and ENCODE).

(a) Horizontal bar plot showing Uhlén[14]/HPA expression categories for cEE host genes across tissue biotypes. Blue indicates low tissue specificity, yellow elevated expression in non-matching tissues, and red elevated expression in the matching tissue.
(b) Same analysis for cEE target genes.
(c) Stacked bar plot comparing category frequencies among cEE host genes, cEE target genes, negative control genes, and all protein-coding genes ($\chi^2 = 2 \times 10^{-66}$, Cramér's V = 0.067).
(d) Violin plots of the Yanai τ tissue-specificity index derived from Uhlén et al. RNA-seq data for the same gene sets. Each data point represents one gene. Statistical significance was assessed using a two-sided Student's t-tests with Bonferroni correction. Violin plots show the distribution of values, with width proportional to data density; central points indicate the median, and the vertical extent represents the full range of observed values.
(e,f) ENCODE RNA-seq expression levels (FPKM; whole-cell, long polyA RNA) for host (e) and target (f) genes of "strong" K-562 cEEs across three cell lines (K-562, A-549, GM12878). Box plots show the median (centre line) and interquartile range (box, 25th to 75th percentiles); whiskers extend to the most extreme values within 1.5×IQR, with points beyond the whiskers plotted as outliers. Differences were assessed using a two-sided Wilcoxon signed-rank test (lowest $p = 8.329 \times 10^{-3}$).

Uhlén categories and τ values reveal a modest but significant shift toward broader expression for cEE-associated genes compared with controls, whereas ENCODE RNA-seq shows a trend toward higher expression in K-562 cells. Together, these orthogonal metrics indicate that most cEEs reside in broadly expressed genes, with a distinct minority exhibiting strong cell- or tissue-specificity.

a

b

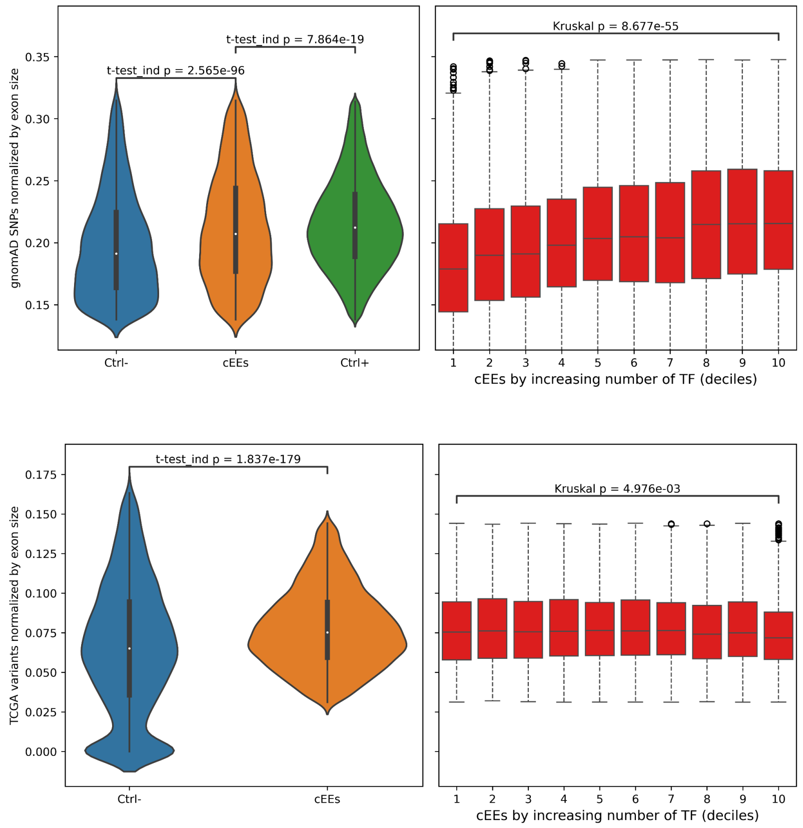

Figure S21: Variant density in cEEs and its relationship to TF-binding load.

(a) Germline variants (gnomAD). Distribution of single-nucleotide polymorphisms (SNPs) from gnomAD within candidate exonic enhancers (cEEs), normalized by exon length. Left panel: SNP density per exon is shown for cEEs (orange; n = 13,481), negative control exons (Ctrl-, blue; n = 13,253), and intergenic enhancers used as positive controls (Ctrl+, green; n = 404,325). Statistical comparisons between groups were performed using a two-sided Student's *t*-test. Right panel: SNP density across cEEs grouped into deciles based on transcription factor (TF) binding density (decile 1 = lowest, decile 10 = highest; equal-sized groups n = 1,348). Statistical significance across deciles was assessed using a Kruskal–Wallis test.

(b) Somatic variants (TCGA). The same analyses applied to tumour-derived single-nucleotide variants (SNVs) from TCGA.

For all panels, each data point represents one exon. Violin plots show the distribution of values, with width proportional to data density; the centre line indicates the median. Box plots show the median (centre line) and interquartile range (box, 25th to 75th percentiles); whiskers extend to the most extreme values within 1.5×IQR, with points beyond the whiskers plotted as outliers. For visualization, values outside the 5th–95th percentile range were removed.

Together, these analyses highlight a distinction between germline and somatic landscapes: germline SNVs accumulate preferentially in the most heavily TF-bound cEEs, consistent with long-term purifying selection, whereas somatic SNVs show a weaker or inverse relationship with TF-binding density, possibly reflecting tumour-specific mutational processes or selective constraints.

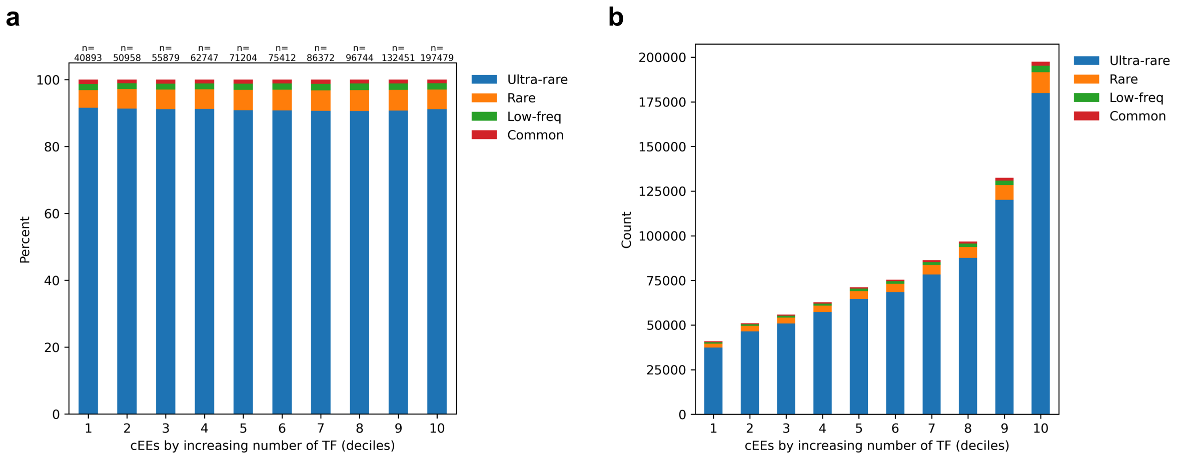

Figure S22: Allele frequency distribution across TF-binding deciles in cEEs.

(a) Stacked bar plots show the proportion of ultra-rare (allele frequency, AF ≤ 0.01%), rare (0.01–0.1%), low-frequency (0.1–1%), and common (AF > 1%) single-nucleotide variants (SNVs) within each transcription factor (TF)-binding decile of candidate exonic enhancers (cEEs), where decile 1 corresponds to the lowest TF-binding density and decile 10 to the highest (n cEEs per decile = 1,348). The allele-frequency composition is similar across deciles (approximately 91% ultra-rare, 6% rare, 2% low-frequency, and 1% common variants). Differences across deciles were assessed using a χ^2 test of independence ($p = 5.76 \times 10^{-14}$; Cramér's V = 0.0068).

(b) The same data displayed as absolute SNV counts per TF-binding decile. Variant counts increase from 41,000 in decile 1 to 198,000 in decile 10, reflecting the larger number of segregating sites in highly TF-bound cEEs, while maintaining a similar skew toward very-low-frequency alleles. Together, these results suggest pervasive purifying selection across the entire cEE set rather than constraint restricted to the most TF-dense elements.

Numbers of variants per decile are indicated above bars in panel (a).

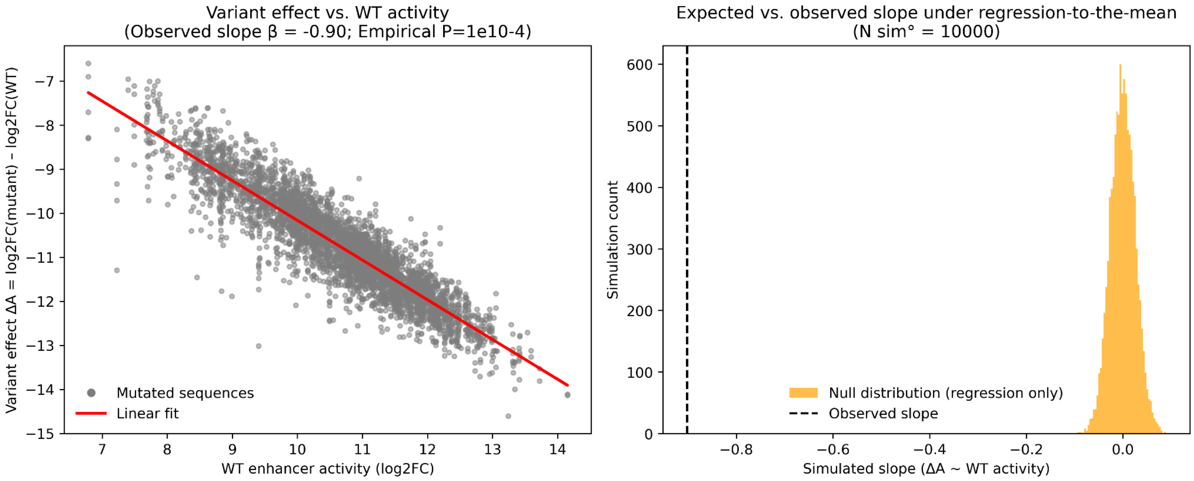

Figure S23: Variant-effect bias is not explained by regression to the mean.

Left: Scatterplot of synonymous-variant effect (ΔA = log2FC_mutant - log2FC_WT) versus wild-type enhancer activity (A_WT) for STARR-seq tested cEEs (n = 4,332; grey dots). The red line shows the least-squares linear fit (observed slope β_obs = -0.90; empirical p = 1×10^{-4}).

Right: Null distribution of regression slopes obtained from 10,000 simulations in which each WT insert was assigned a pseudo-variant effect drawn from a normal distribution $N(0, \sigma^2)$, where σ^2 corresponds to the empirical variance of observed variant effects. The dashed line indicates the observed slope (β_obs). The observed slope lies outside the null distribution, indicating that the negative correlation is not explained by regression-to-the-mean effects alone.

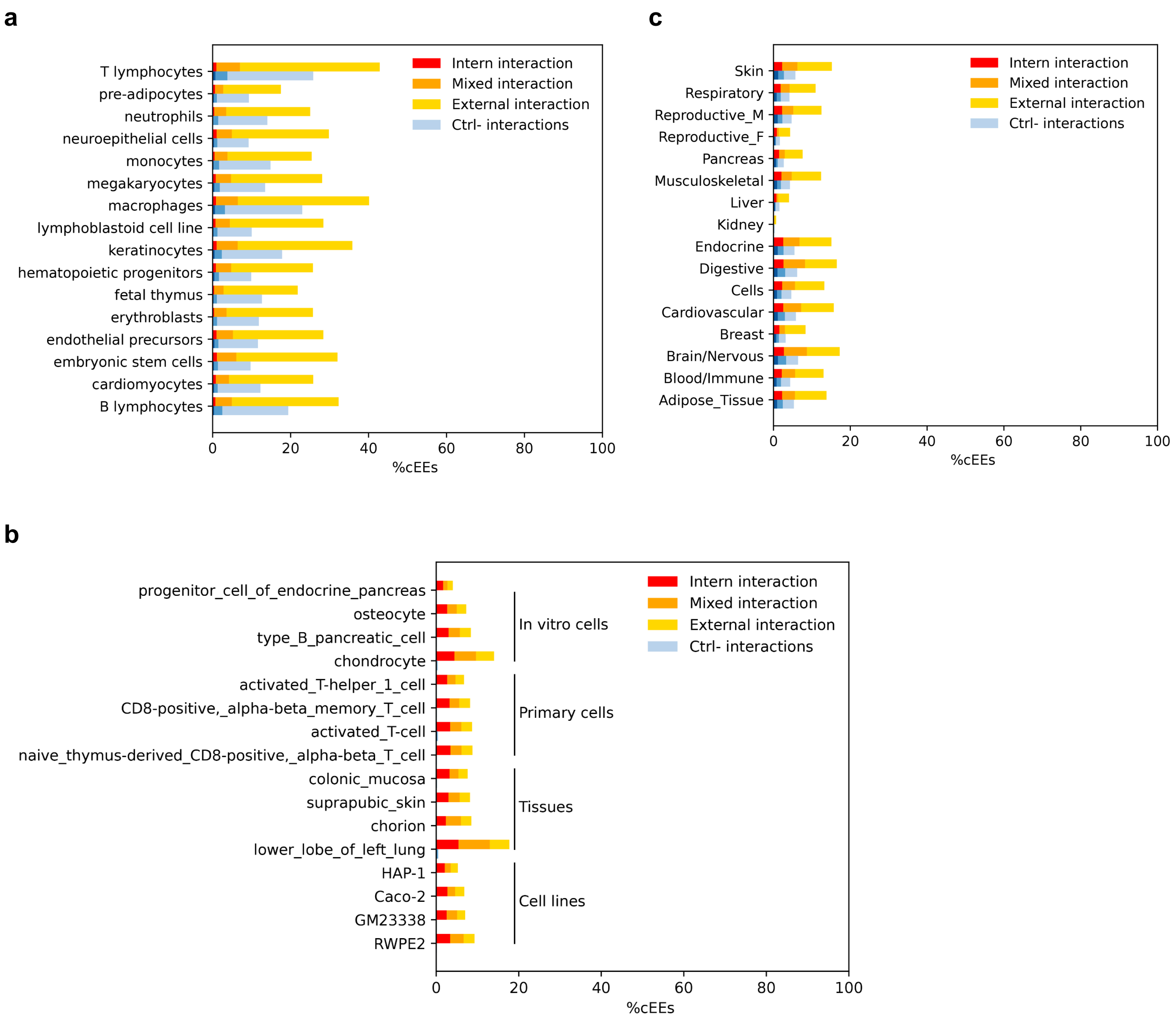

Figure S24: Interaction landscape of exon enhancers across cellular and tissue contexts.

Candidate Exon Enhancers (cEEs) exhibit distinct chromatin interactions with target genes, categorized as internal (within the host gene, red), external (distal genes, yellow), or mixed (host + distal interactions, orange). For each dataset, values represent the percentage of cEEs showing each interaction category, normalized to the total number of cEEs. **(a)** Normalized pCap-HiC interactions from Laverré et al.[5] reveal the prevalence of these interaction types across diverse human cell lines, highlighting variations in cEEs connectivity depending on the cellular context. **(b)** ENCODE-E2G[15] interaction data show the four most frequent cEEs interactions, classified into *in vitro* cells, primary cells, tissues, and cell lines. **(c)** GTEx eQTL associations link cEEs to their target genes across 54 human tissues, normalized into 16 biotypes following the framework of de Langen et al.[16] (*Cell Genom.* 2023).

Bars indicate the proportion of cEEs per interaction category. Light blue bars represent control exon interactions (Ctrl-), defined as coding exons without enhancer activity. No statistical testing was performed, as the figure is intended to provide a descriptive overview of interaction patterns across datasets.

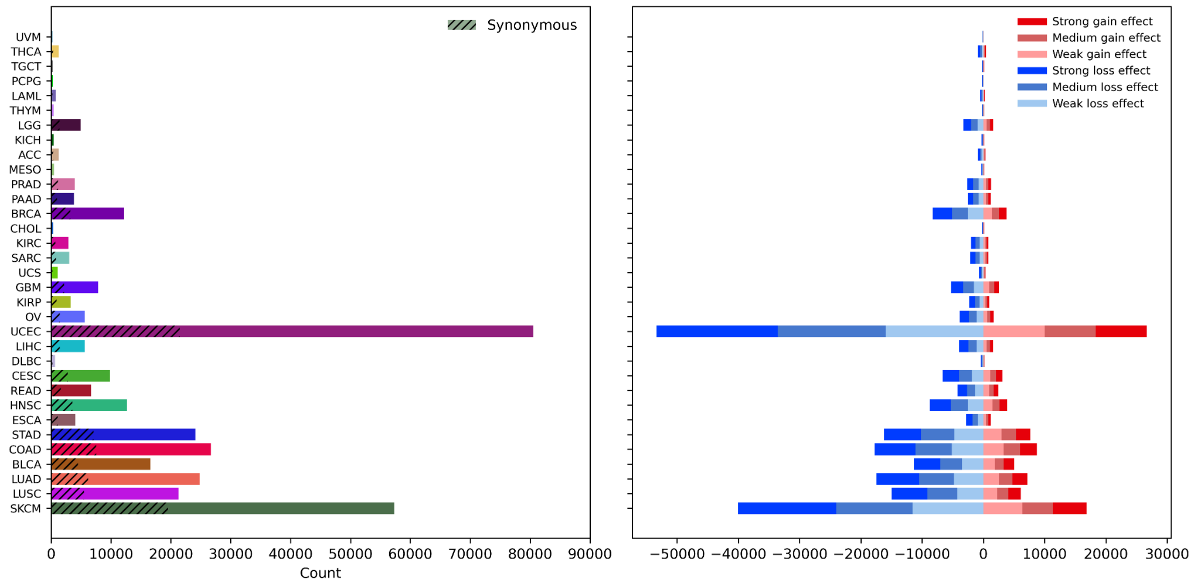

Figure S25: PanCancerAtlas variants in cEEs and their impact on transcription factor binding.

Distribution and predicted regulatory impact of single-nucleotide polymorphisms (SNPs) from the PanCancer Atlas overlapping candidate exonic enhancers (cEEs). SNP coordinates were mapped to the hg19 reference genome using UCSC liftOver. **Left panel:** Number of unique somatic SNPs overlapping cEEs for each cancer type. Each bar corresponds to one cancer type, and bar height indicates the total number of SNPs identified. Hatched segments denote synonymous variants. **Right panel:** Predicted effects of SNPs on transcription factor binding sites (TFBSs), assessed using the FABIAN-variant tool with JASPAR motifs. SNPs are classified according to the predicted direction (gain, red; loss, blue) and magnitude of effect (strong, medium, or weak, indicated by colour intensity). Values represent counts of SNPs per cancer type in each effect category. This analysis suggests that somatic mutations in cEEs may alter transcription factor binding, potentially contributing to regulatory changes across different cancer types.

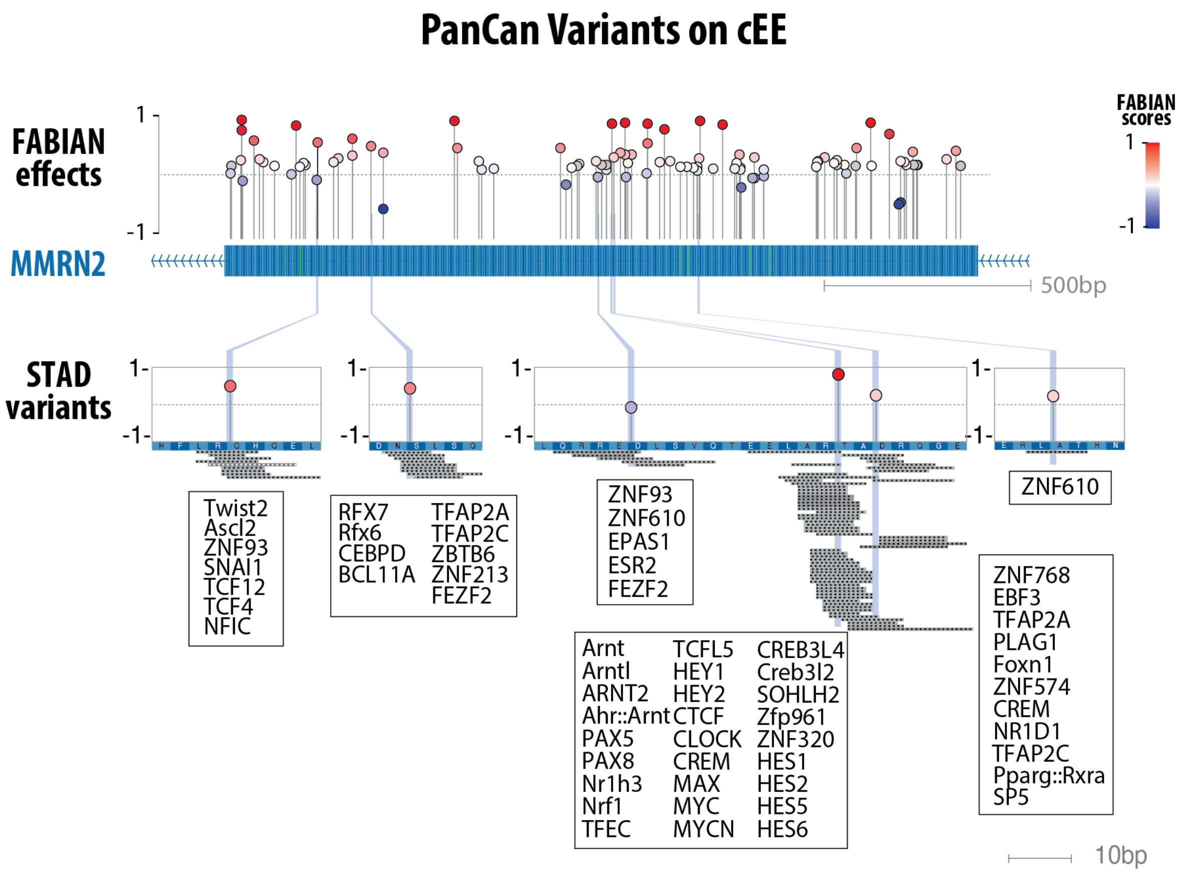

Figure S26: PanCancerAtlas lollipop genomic track.

Example genomic track at the *MMRN2* locus illustrating predicted transcription factor (TF) binding changes for cancer-associated variants identified in the PanCancer Atlas. Each lollipop represents one single-nucleotide variant, with the lollipop height and colour indicating the predicted effect on TF binding affinity based on FABIAN-variant[17] scores (red, increased binding affinity; blue, decreased binding affinity; score range -1 to +1).

Shown are stomach adenocarcinoma (STAD) variants overlapping a candidate exonic enhancer (cEE), which coincide with multiple JASPAR TF motif sites (listed). TF motifs predicted to be affected by the variants are indicated below the track.

This panel is shown as a representative example. A UCSC Genome Browser public track hub containing all FABIAN-annotated PanCancer variants overlapping cEEs is available via the UCSC Genome Browser public hub listings.

The complete set of variants and predicted TF binding effects is provided in the corresponding UCSC public track hub: https://genome.ucsc.edu/cgi-bin/hgHubConnect.

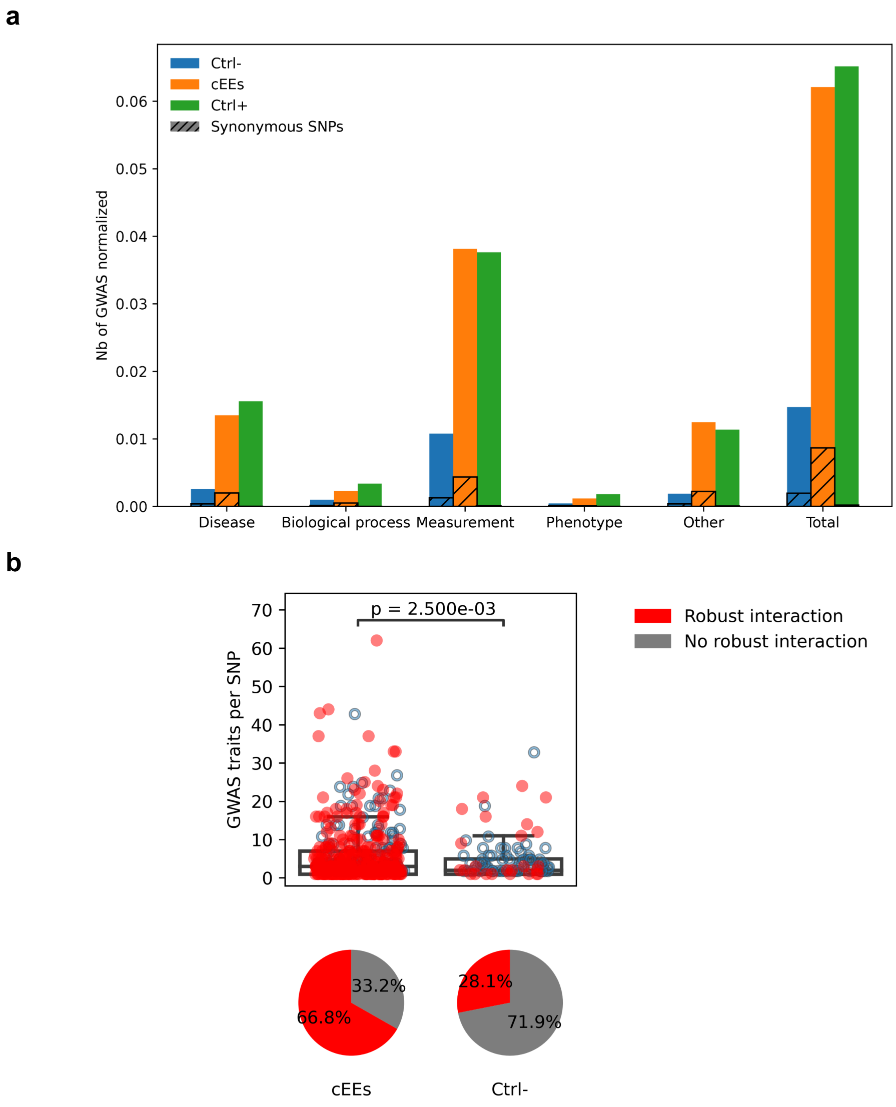

Figure S27: GWAS Catalog Variants in candidate Exonic Enhancers (cEEs).

(a) Overlap of NHGRI-EBI GWAS catalog[18] single-nucleotide polymorphisms (SNPs; linkage disequilibrium $r^2 > 0.8$) with candidate exonic enhancers (cEEs). GWAS traits were mapped into five parent categories (Disease, Biological process, Measurement, Phenotype, and Other). For each category, bars indicate the number of GWAS SNPs overlapping cEEs (total SNPs overlapping cEEs: n = 837), normalized by the total number of elements in each dataset (cEEs: n = 13,481; negative control exons (Ctrl-): n = 13,253; positive control intergenic enhancers (Ctrl+): n = 404,325). Negative controls show a lower overlap (total SNPs: n = 195), whereas overlap rates for cEEs are comparable to positive control enhancers (total SNPs: n = 26,347). Hatched bars indicate synonymous GWAS SNPs overlapping cEEs. **(b)** Pleiotropic GWAS SNPs (from Watanabe *et al.* 2019) overlapping cEEs or control exons. Each data point represents one GWAS SNP, with the y-axis indicating the number of associated traits per SNP. SNPs located in exons showing robust regulatory interactions (supported by concordant pCap-HiC, ENCODE-rE2G, and GTEx eQTL evidence) are shown in red; SNPs without robust interactions are shown in grey. Box plots summarize the distributions (median, interquartile range; whiskers extend to 1.5×IQR). Pie charts indicate the proportion of SNPs with (red) or without (grey) robust interactions in cEEs and Ctrl- datasets. Statistical significance was assessed using a two-sided Anderson–Darling test (p = 0.0025).

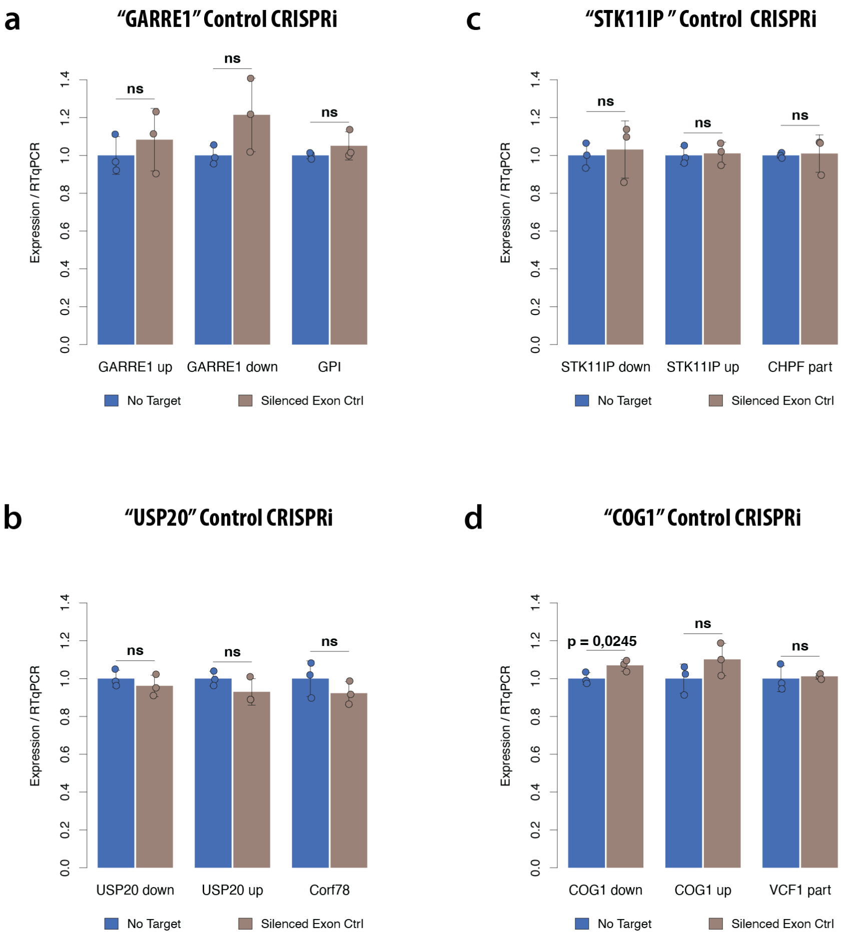

Figure S28: CRISPRi Exon controls.

RT–qPCR analysis of gene expression following CRISPRi targeting of control exons (non-enhancer coding regions) within four genes used in the main CRISPRi experiments:
(a) *GARRE1* exon control (chr19:34327989–34328151),
(b) *USP20* exon control (chr9:129863185–129863299),
(c) *STK11IP* exon control (chr2:219608046–219608430), and
(d) *COG1* exon control (chr17:73201108–73201900).
Each panel shows relative mRNA expression levels of the host gene (upstream and downstream exons) and one neighbouring gene, normalized to the non-targeting control. Bars represent mean ± s.e.m. across biological replicates (n = 3). Individual data points indicate biological replicates. Statistical significance was assessed using a one-tailed Student's *t*-test. No significant expression changes were observed upon silencing these control exons (ns, COG1 down p = 0,0245), confirming that the transcriptional effects reported in Fig. 6 result from specific inactivation of exonic enhancers rather than non-specific CRISPRi activity.

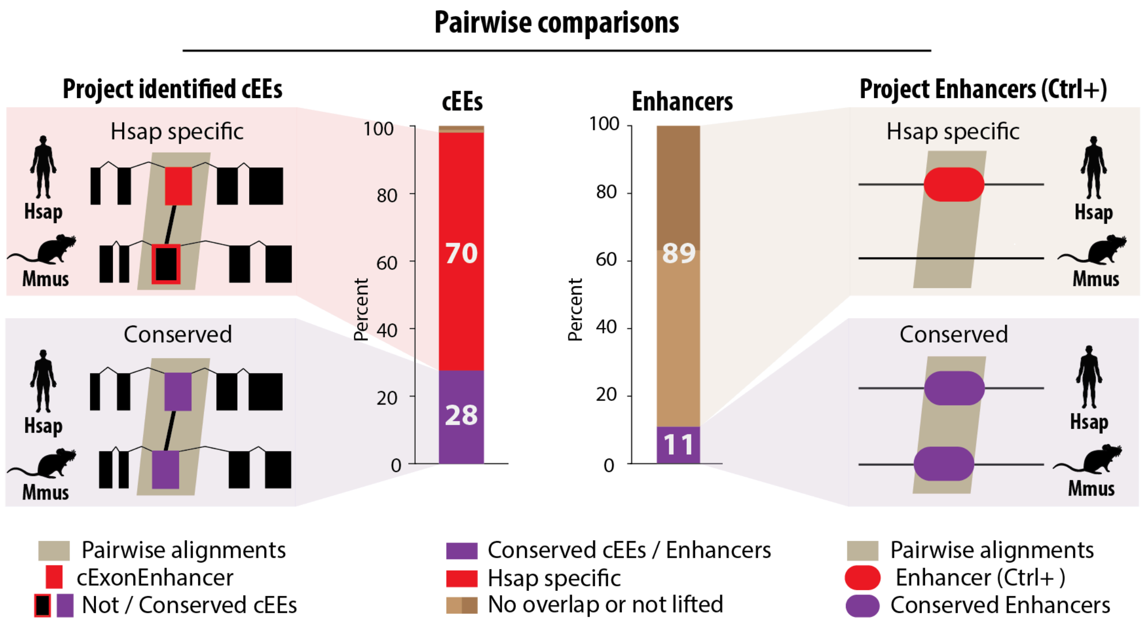

Figure S29: Comparative conservation of exonic and intergenic enhancers between human and mice.

Pairwise conservation of candidate exonic enhancers (cEEs) and intergenic enhancers (Ctrl+) between *Homo sapiens* (*H.sap*) and *Mus musculus* (*M.mus*) using LastZ-net pairwise alignments. **Left panel (cEEs):** The proportion of cEEs classified as *H.sap*-specific (70%) versus conserved between both species (28%). **Right panel (Enhancers):** Comparison with ENCODE intergenic enhancers used as positive controls, showing a lower conservation rate (11%) compared to cEEs (28%). Annotations; Red: *H.sap*-specific cEEs or enhancers. Purple: Conserved cEEs or enhancers. Beige: Pairwise alignments between species. These results suggest that a larger fraction of cEEs are evolutionarily conserved compared to classical intergenic enhancers, highlighting their potential functional significance. Percentages indicate the fraction of elements relative to the total number analysed (cEEs: n = 13,481; ENCODE intergenic enhancers (Ctrl+): n = 404,325).

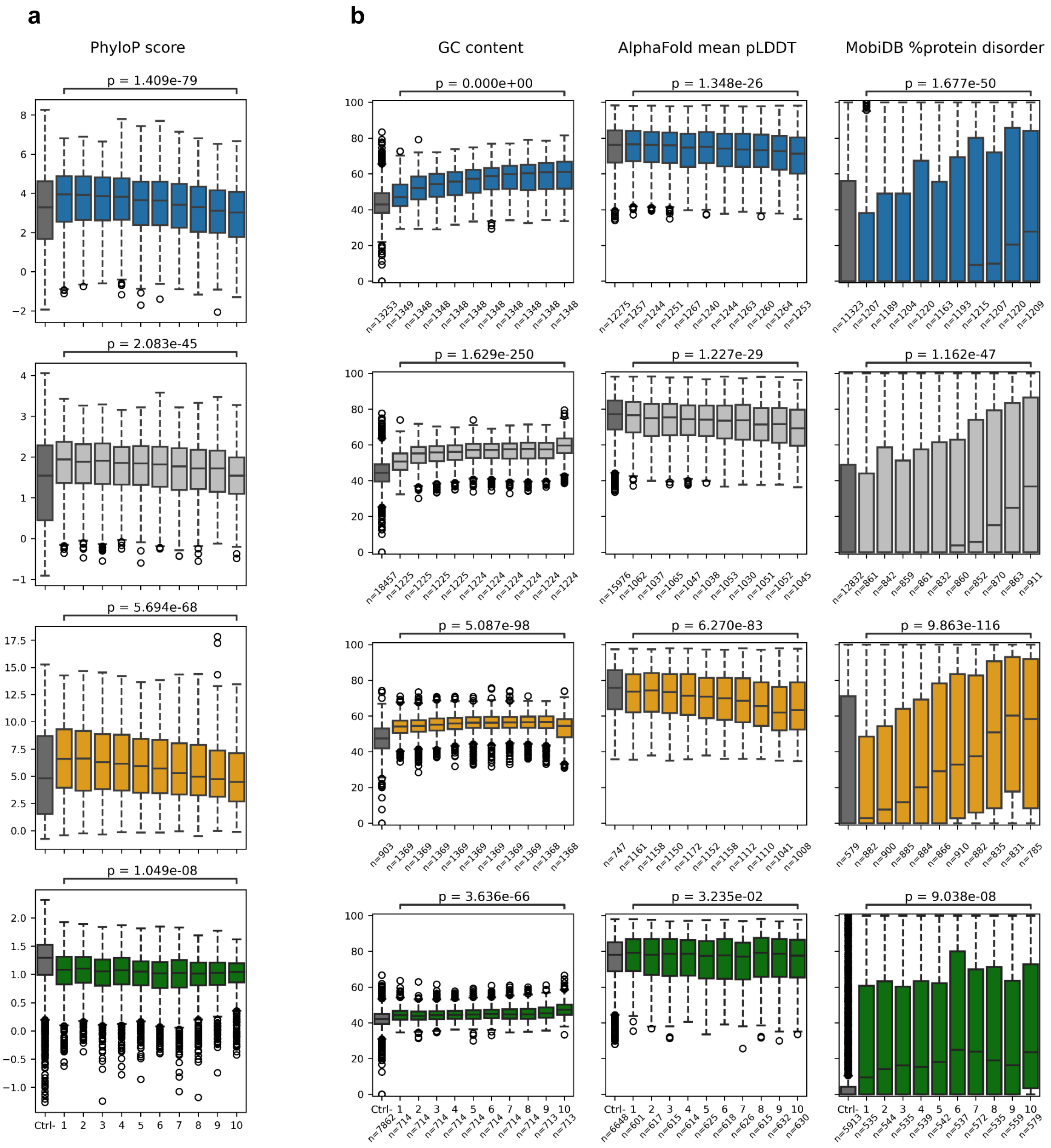

Figure S30: Evolutionary conservation and structural properties of exonic enhancers.

Conservation and structural characteristics of candidate exonic enhancers (cEEs), grouped by transcription factor (TF) binding density deciles, across four species: *Homo sapiens* (blue; n = 13,481 cEEs), *Mus musculus* (gray, n = 12,244), *Drosophila melanogaster* (orange, n = 13,688), and *Arabidopsis thaliana* (green, n = 7,138).

(a) Evolutionary conservation: PhyloP scores[19] represent evolutionary constraints across species, with higher scores indicating stronger conservation. PhyloP scores are derived from multiple genome alignments: 100 species for *H.sap*, 35 species for *M.mus*, 124 species for *D.mel*, and 63 species for *A.tha*. N per decile group: *H.sap* n = 1,348; *M.mus* n = 1,225; *D.mel* n = 1,369; *A.tha* n = 714.

(b) Structural characteristics of cEEs:

- GC content: The proportion of guanine-cytosine nucleotides in cEE sequences, analyzed across TF-binding deciles.
- AlphaFold[20] mean pLDDT scores: Predicted protein structure confidence scores for cEEs, with higher values reflecting more stable structural domains.

- MobiDB[21] % protein disorder: Proportion of intrinsically disordered protein regions in cEEs, suggesting increased flexibility in TF-bound exons.

In all panels, each data point represents one cEE, grouped by TF-binding density deciles. Statistical comparisons across TF-binding deciles were performed separately for each species using the Kruskal–Wallis test.

Comparisons revealed significant differences in evolutionary conservation, nucleotide composition, and structural properties of cEEs across TF-binding densities. Box plots show the median (centre line) and interquartile range (box, 25th to 75th percentiles); whiskers extend to the most extreme values within 1.5×IQR, with points beyond the whiskers plotted as outliers.

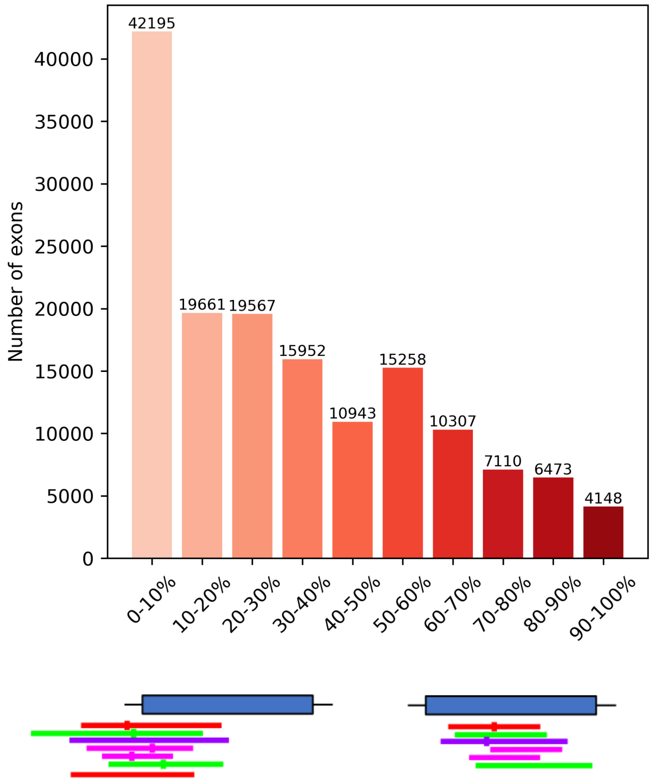

Figure S31: Selection of exonic enhancers based on transcription factor summit density.

Distribution of transcription factor (TF) ChIP-seq summits relative to TF number in *Homo sapiens* merged protein-coding exons. Top panel: Histogram displaying the number of exons (y-axis) categorized by TF ChIP-seq summit-to-TF number ratios (x-axis) in percentage bins. Most exons fall within the 0–10% range, with a progressively lower number of exons observed in higher TF ChIP-seq summit ratio bins. Bottom panel: Schema visualization of TF ChIP-seq summit distributions across selected exons, illustrating variance and thresholds for defining candidate exonic enhancers (cEEs). To ensure stringent selection criteria, only exons with a TF ChIP-seq summit-to-TF number ratio greater than 50 were retained for cEEs classification.

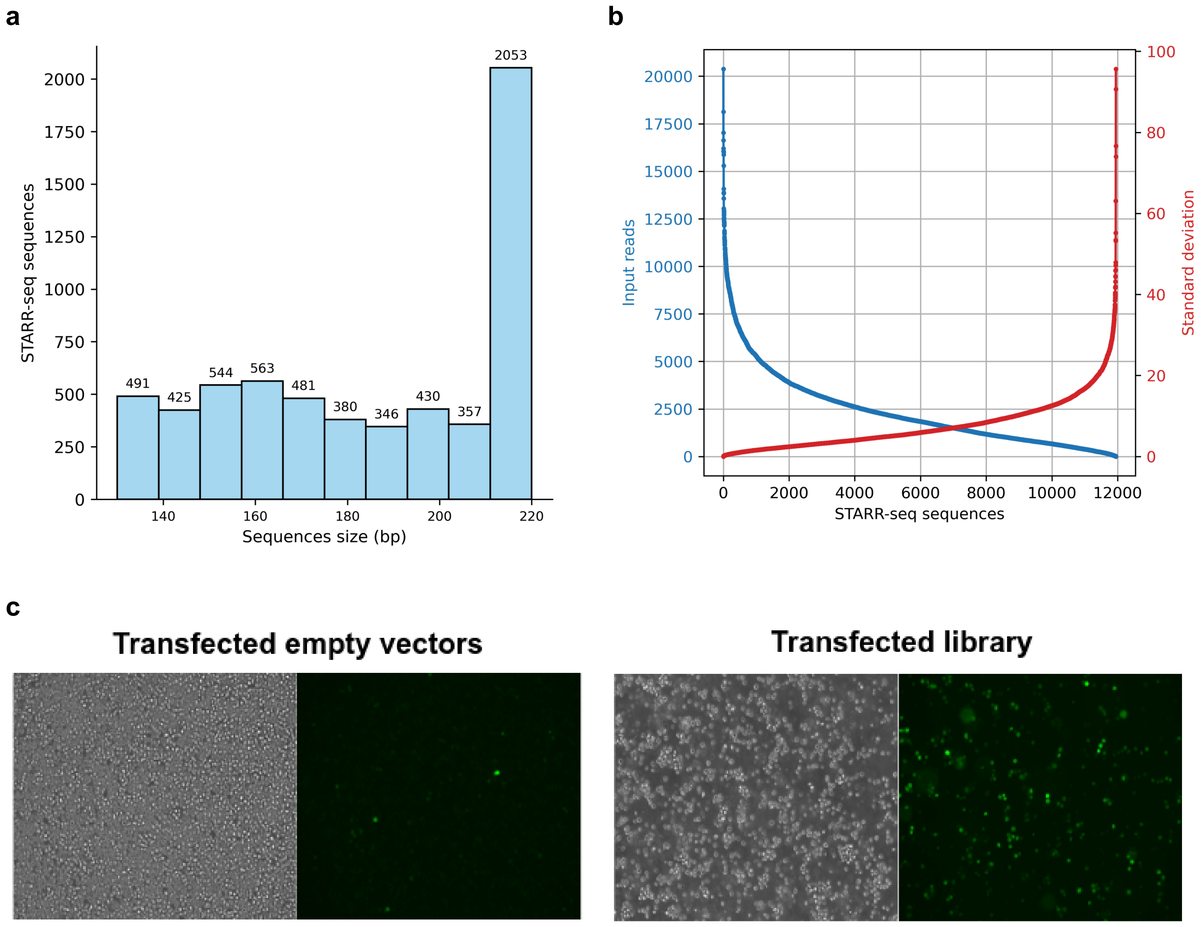

Figure S32: STARR-seq experimental design and validation of exonic enhancer activity.

Overview of the STARR-seq experimental setup, including sequence characteristics, input read distributions, and transfection results.

(a) Sequence size distribution: Histogram showing the distribution of tested sequence lengths (in base pairs, bp). Most sequences are between 140–220 bp, with a peak at 220 bp.

(b) Input read distribution and standard deviation: The number of input reads (blue curve) and the standard deviation (red curve) across STARR-seq sequences. Each point corresponds to one STARR-seq sequence, ranked by input read count.

(c) Transfection efficiency: Fluorescence microscopy images comparing cells transfected with empty STARR-seq vector (left) versus the exonic enhancer STARR-seq library (right). The transfected library shows a significantly higher number of fluorescent cells, indicating successful expression from enhancer-active sequences. These data validate the selection and functional testing of candidate exonic enhancers using STARR-seq. Images are representative of five biological experiments with similar results.

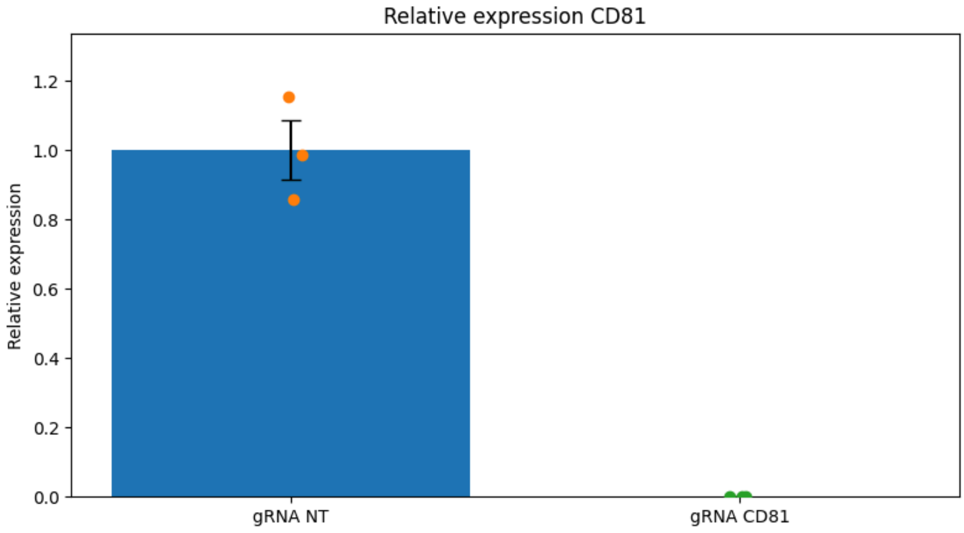

Figure S33: Validation of CRISPRi-competent K-562 cells by inhibition of CD81 expression.

Validation of CRISPRi-mediated repression in K-562 cells stably expressing dCas9–KRAB–MeCP2 was performed with specific guide RNA targeting the CD81 promoter (gRNA CD81) and selected with puromycin. CD81 expression was measured by RT-qPCR and is presented relative to the non-targeting control. The marked reduction in CD81 mRNA confirms efficient CRISPRi knockdown in the dCas9–KRAB–MeCP2 cell line. Data are shown as mean ± s.e.m. across biological replicates (n = 3).

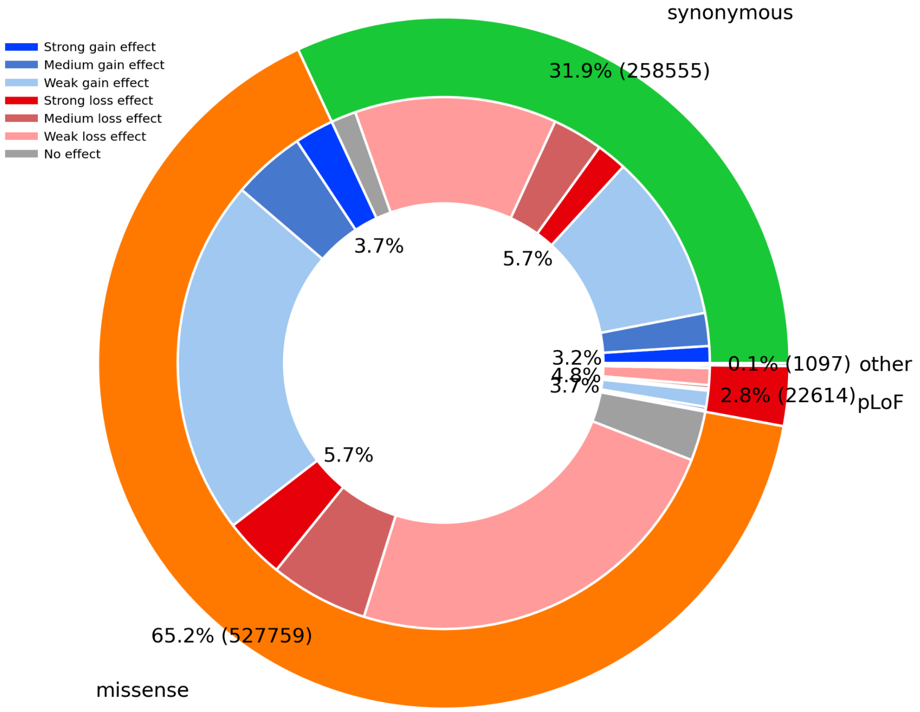

Figure S34: Distribution and regulatory impact of gnomAD SNPs in exonic enhancers.

This figure illustrates the distribution of gnomAD v.3 SNPs within candidate exonic enhancers (cEEs) and their predicted effects on transcription factor (TF) binding, as assessed by the FABIAN-variant tool. The analysis considers SNPs overlapping a ReMap TF peak and its corresponding transcription factor binding site (TFBS) from JASPAR.

Outer ring: Proportion of SNP types found in cEEs, including synonymous (green, 31.9%), missense (orange, 65.2%), pLoF (predicted loss-of-function, red, 2.8%), and other variants (grey, 0.1%). Numbers shown in parentheses next to each category indicate the absolute number of variants.

Inner ring: TF-binding disruption effects of SNPs, with blue shades indicating gain of TF binding and red shades indicating loss of TF binding. Each effect is further classified into three categories based on disruption scores: Strong (>0.66, dark shades), Medium (0.33–0.66, mid shades), and Weak (>0 but <0.33, light shades). Only the best score is taken into account. Gray represents SNPs with no TF/TFBS overlap.

These results show that a fraction of missense and synonymous SNPs within cEEs significantly disrupt TF binding, with both loss and gain effects, highlighting the potential regulatory consequences of genetic variation within exonic enhancers.

References

1. Karczewski, K. J. *et al.* The mutational constraint spectrum quantified from variation in 141,456 humans. *Nature* **581**, 434–443 (2020).

2. Hoadley, K. A. *et al.* Cell-of-Origin Patterns Dominate the Molecular Classification of 10,000 Tumors from 33 Types of Cancer. *Cell* **173**, 291-304.e6 (2018).

3. Birnbaum, R. Y. *et al.* Systematic Dissection of Coding Exons at Single Nucleotide Resolution Supports an Additional Role in Cell-Specific Transcriptional Regulation. *PLOS Genet.* **10**, e1004592 (2014).

4. Forrest, A. R. R. *et al.* A promoter-level mammalian expression atlas. *Nature* **507**, 462–470 (2014).

5. Andersson, R. *et al.* An atlas of active enhancers across human cell types and tissues. *Nature* **507**, 455–461 (2014).

6. Hammal, F., de Langen, P., Bergon, A., Lopez, F. & Ballester, B. ReMap 2022: a database of Human, Mouse, Drosophila and Arabidopsis regulatory regions from an integrative analysis of DNA-binding sequencing experiments. *Nucleic Acids Res.* **50**, D316–D325 (2022).

7. Sheffield, N. C. & Bock, C. LOLA: Enrichment analysis for genomic region sets and regulatory elements in R and Bioconductor. *Bioinformatics* **32**, 587–589 (2015).

8. Meylan, P., Dreos, R., Ambrosini, G., Groux, R. & Bucher, P. EPD in 2020: enhanced data visualization and extension to ncRNA promoters. *Nucleic Acids Res.* **48**, D65–D69 (2020).

9. Rodriguez, J. M. *et al.* APPRIS: selecting functionally important isoforms. *Nucleic Acids Res.* **50**, D54–D59 (2022).

10. ENCODE Project Consortium *et al.* Expanded encyclopaedias of DNA elements in the human and mouse genomes. *Nature* **583**, 699–710 (2020).

11. Zou, Z., Ohta, T. & Oki, S. ChIP-Atlas 3.0: a data-mining suite to explore chromosome architecture together with large-scale regulome data. *Nucleic Acids Res.* **52**, W45–W53 (2024).

12. Tian, F., Yang, D.-C., Meng, Y.-Q., Jin, J. & Gao, G. PlantRegMap: charting functional regulatory maps in plants. *Nucleic Acids Res.* **48**, D1104–D1113 (2020).

13. Castro-Mondragon, J. A. *et al.* JASPAR 2022: the 9th release of the open-access database of transcription factor binding profiles. *Nucleic Acids Res.* **50**, D165–D173 (2022).

14. Uhlen, M. *et al.* A genome-wide transcriptomic analysis of protein-coding genes in human blood cells. *Science* **366**, eaax9198 (2019).

15. Gschwind, A. R. *et al.* An encyclopedia of enhancer-gene regulatory interactions in the human genome. *BioRxiv Prepr. Serv. Biol.* 2023.11.09.563812 (2023) doi:10.1101/2023.11.09.563812.

16. de Langen, P. *et al.* Characterizing intergenic transcription at RNA polymerase II binding sites in normal and cancer tissues. *Cell Genomics* **3**, 100411 (2023).

17. Steinhaus, R., Robinson, P. N. & Seelow, D. FABIAN-variant: predicting the effects of DNA variants on transcription factor binding. *Nucleic Acids Res.* **50**, W322–W329 (2022).

18. Sollis, E. *et al.* The NHGRI-EBI GWAS Catalog: knowledgebase and deposition resource. *Nucleic Acids Res.* **51**, D977–D985 (2023).

19. Perez, G. *et al.* The UCSC Genome Browser database: 2025 update. *Nucleic Acids Res.* **53**, D1243–D1249 (2025).

20. Jumper, J. *et al.* Highly accurate protein structure prediction with AlphaFold. *Nature* **596**, 583–589 (2021).

21. Piovesan, D. *et al.* MOBIDB in 2025: integrating ensemble properties and function annotations for intrinsically disordered proteins. *Nucleic Acids Res.* **53**, D495–D503 (2025).
