# Supplementary material for: Exonic enhancers are a widespread class of dual-function regulatory elements
Source: Nat Commun. 2026 Apr 2;17:4755. doi: 10.1038/s41467-026-71220-6 (PMC13216554; doi:10.1038/s41467-026-71220-6)
Supplement: Supplementary file 2 — Description of Additional Supplementary Files [file 41467_2026_71220_MOESM2_ESM.pdf]

Description of Additional Supplementary Files

Supplementary Data 1
Multi-omics integrative tables used to characterise cEEs (motif load, chromatin accessibility, TF binding metrics, eQTL overlaps, conservation and sequence features). Provides the numeric values underlying genome-scale plots.

Supplementary Data 2
Per-element activity tables from STARR-seq for candidate cEEs, including raw/normalised counts, log2(cDNA/gDNA), significance (P, FDR), and pass/fail flags used to define active elements and variants.

Supplementary Data 3
Experimental metadata for STARR-seq: construct annotations, library IDs, sample sheets, sequencing run information, replicate mapping, and QC summaries (read depth, mapping %, duplicate rate).

Supplementary Data 4
DESeq2 outputs for TCGA comparisons relevant to cEEs (e.g., gene expression contrasts between groups) with log2 fold-changes, standard errors, Wald statistics, P values and BH-adjusted q-values; includes sample/group mappings used for each comparison.

Supplementary Data 5
Compressed archive of BED files constituting the exonic enhancer (cEE) catalogue across species. Coordinates are provided per species/genome build with minimal columns (chrom, start, end, ID, score/strand where available). Intended for direct loading into genome browsers (UCSC/IGV) and for overlap/enrichment analyses.
