# Supplementary material for: Exonic enhancers are a widespread class of dual-function regulatory elements
Source: Nat Commun. 2026 Apr 2;17:4755. doi: 10.1038/s41467-026-71220-6 (PMC13216554; doi:10.1038/s41467-026-71220-6)
Supplement: Supplementary file 8 — Reporting Summary [file 41467_2026_71220_MOESM8_ESM.pdf]

nature portfolio

Corresponding author(s): Benoit Ballester
Last updated by author(s): 08/01/2026

Reporting Summary

Nature Portfolio wishes to improve the reproducibility of the work that we publish. This form provides structure for consistency and transparency in reporting. For further information on Nature Portfolio policies, see our Editorial Policies and the Editorial Policy Checklist.

Statistics

For all statistical analyses, confirm that the following items are present in the figure legend, table legend, main text, or Methods section.

n/a	Confirmed	
☐	☒	The exact sample size (*n*) for each experimental group/condition, given as a discrete number and unit of measurement
☐	☒	A statement on whether measurements were taken from distinct samples or whether the same sample was measured repeatedly
☐	☒	The statistical test(s) used AND whether they are one- or two-sided<br>*Only common tests should be described solely by name; describe more complex techniques in the Methods section.*
☐	☒	A description of all covariates tested
☐	☒	A description of any assumptions or corrections, such as tests of normality and adjustment for multiple comparisons
☐	☒	A full description of the statistical parameters including central tendency (e.g. means) or other basic estimates (e.g. regression coefficient) AND variation (e.g. standard deviation) or associated estimates of uncertainty (e.g. confidence intervals)
☐	☒	For null hypothesis testing, the test statistic (e.g. *F*, *t*, *r*) with confidence intervals, effect sizes, degrees of freedom and *P* value noted<br>*Give P values as exact values whenever suitable.*
☒	☐	For Bayesian analysis, information on the choice of priors and Markov chain Monte Carlo settings
☒	☐	For hierarchical and complex designs, identification of the appropriate level for tests and full reporting of outcomes
☐	☒	Estimates of effect sizes (e.g. Cohen's *d*, Pearson's *r*), indicating how they were calculated

Our web collection on statistics for biologists contains articles on many of the points above.

Software and code

Policy information about availability of computer code

Data collection	Data supporting the findings of this study are available in a Zenodo repository (https://doi.org/10.5281/zenodo.17208730), organized by analysis block in accordance with the structure of the paper. All datasets used in this study are publicly available or have been deposited in appropriate repositories.<br>No custom software was used during data acquisition beyond vendor-supplied instrument control software. Sequencing was run on an Illumina NextSeq 2000 using the manufacturer's control software; qPCR data were acquired on a Thermo Fisher QuantStudio™ 6 Flex with the instrument software. Dual-luciferase readings and cytometry acquisitions were recorded with the instruments' native software; see Methods for instrument details.<br>Newly generated experimental data in this study comprise STARR-seq libraries, luciferase reporter assays, qRT-PCR, CRISPRi perturbations and flow-cytometry/plate-reader measurements performed on K-562 cells; sequencing was run on an Illumina NextSeq 2000 (manufacturer control software) and instrument files were captured with vendor software. Newly generated STARR-seq data are deposited in GEO (GSE292804) and processed/derived files are archived on Zenodo (https://doi.org/10.5281/zenodo.17208730). In addition, we re-analysed large public catalogues and summary data to build the exon-enhancer catalogue: ReMap (catalogue version and release — see Methods and Zenodo), ENCODE/ChIP-Atlas peak sets, GENCODE/Ensembl gene models, FANTOM5 TSS, GTEx v8 eQTL and TCGA PanCanAtlas summary data. For each reused resource we cite the release/version and accession(s) in Methods and Data Availability; selection and QC filters are described in Methods.
Data analysis	All code and software is referenced in the manuscript and in the Zenodo repository. Code and bioinformatics environments are available on GitHub (https://github.com/benoitballester/ExonEnhancer). Processed data and files are available in the Zenodo repository https://doi.org/10.5281/zenodo.17208730). Both the data and code are publicly available for replication of the entire study.<br>All analyses were performed with open-source tools and custom Python/R scripts available at GitHub and archived on Zenodo (https://doi.org/10.5281/zenodo.17208730); exact dependencies are pinned in the repository environment files. Core tools included: Bowtie2 v2.5.1,

SAMtools v1.18, Sambamba v1.0.0, MACS2 v2.2.7.1, BEDTools v2.30.0, khmer (unique-kmers.py), UCSC Kent utilities (bigWigAverageOverBed v2), liftOver, deepTools, pyDESeq2 v0.4.8, the dnds Python package (Nei–Gojobori), and the geno2proteo R package v0.0.6. For design/annotation steps we used CRISPOR (sgRNA design) and FABIAN-variant (TF-binding impact). Statistical analyses used two-sided tests as indicated (Mann–Whitney, Kruskal–Wallis, χ^2, Wald) with Benjamini–Hochberg FDR control; full parameterization is detailed in Methods and the code repository.

For manuscripts utilizing custom algorithms or software that are central to the research but not yet described in published literature, software must be made available to editors and reviewers. We strongly encourage code deposition in a community repository (e.g. GitHub). See the Nature Portfolio guidelines for submitting code & software for further information.

Data

Policy information about availability of data

All manuscripts must include a data availability statement. This statement should provide the following information, where applicable:

- Accession codes, unique identifiers, or web links for publicly available datasets
- A description of any restrictions on data availability
- For clinical datasets or third party data, please ensure that the statement adheres to our policy

Data availability. Data supporting the findings of this study are available in Zenodo under the identifier https://doi.org/10.5281/zenodo.17208730, organized by analysis block as described in the Methods. Newly generated STARR-seq data have been deposited in GEO under accession GSE292804 and will be made public upon publication (editorial access provided to reviewers during peer review). Public resources used in the study include genome annotations (UCSC/Ensembl), DNase-seq/ATAC-seq and ChIP-seq datasets (ENCODE, ChIP-Atlas, PlantRegMap), TSS catalogs (FANTOM5, modENCODE, Arabidopsis), promoter capture Hi-C, GTEx v8 eQTLs, TCGA PanCanAtlas summary data, and multi-species phyloP conservation tracks; all are available from their respective repositories and are cited in the manuscript. A UCSC public track hub listing the exonic enhancers identified here is provided for interactive visualization. No restrictions apply to data availability. Source Data are provided with this paper.

Research involving human participants, their data, or biological material

Policy information about studies with human participants or human data. See also policy information about sex, gender (identity/presentation), and sexual orientation and race, ethnicity and racism.

Reporting on sex and gender	*Use the terms sex (biological attribute) and gender (shaped by social and cultural circumstances) carefully in order to avoid confusing both terms. Indicate if findings apply to only one sex or gender; describe whether sex and gender were considered in study design; whether sex and/or gender was determined based on self-reporting or assigned and methods used. Provide in the source data disaggregated sex and gender data, where this information has been collected, and if consent has been obtained for sharing of individual-level data; provide overall numbers in this Reporting Summary. Please state if this information has not been collected. Report sex- and gender-based analyses where performed, justify reasons for lack of sex- and gender-based analysis.*
Reporting on race, ethnicity, or other socially relevant groupings	*Please specify the socially constructed or socially relevant categorization variable(s) used in your manuscript and explain why they were used. Please note that such variables should not be used as proxies for other socially constructed/relevant variables (for example, race or ethnicity should not be used as a proxy for socioeconomic status). Provide clear definitions of the relevant terms used, how they were provided (by the participants/respondents, the researchers, or third parties), and the method(s) used to classify people into the different categories (e.g. self-report, census or administrative data, social media data, etc.) Please provide details about how you controlled for confounding variables in your analyses.*
Population characteristics	*Describe the covariate-relevant population characteristics of the human research participants (e.g. age, genotypic information, past and current diagnosis and treatment categories). If you filled out the behavioural & social sciences study design questions and have nothing to add here, write "See above."*
Recruitment	*Describe how participants were recruited. Outline any potential self-selection bias or other biases that may be present and how these are likely to impact results.*
Ethics oversight	*Identify the organization(s) that approved the study protocol.*

Note that full information on the approval of the study protocol must also be provided in the manuscript.

Field-specific reporting

Please select the one below that is the best fit for your research. If you are not sure, read the appropriate sections before making your selection.

☒ Life sciences ☐ Behavioural & social sciences ☐ Ecological, evolutionary & environmental sciences

For a reference copy of the document with all sections, see nature.com/documents/nr-reporting-summary-flat.pdf

Life sciences study design

All studies must disclose on these points even when the disclosure is negative.

Sample size	No statistical method was used to predetermine sample size. For plate-based assays (luciferase; qPCR; CRISPRi readouts), we used ≥3

Sample size	biological replicates (See methods). For STARR-seq, we prepared ≥3 independent transfections/libraries and sequenced to depth sufficient to quantify activity across all elements (see Methods). These sizes follow community standards and our prior/pilot experiments, which provide power to detect ~1.5–2× effects with typical variance in these assays.
Data exclusions	No ad-hoc exclusions were made. Pre-specified QC criteria were applied uniformly:<br>Luciferase/qPCR: wells with failed controls, saturation, or Renilla/housekeeper outliers (>3 SD from plate median) were excluded.<br>Flow cytometry (if applicable): samples with <85% viability, <5,000 events, or failed compensation were excluded.<br>STARR-seq: reads failing mapping/QC, PCR duplicates, and features with insufficient counts (low-abundance filters defined in Methods) were removed.<br>All criteria were defined a priori in the analysis notebooks.
Replication	All key findings were reproduced in independent biological replicates with consistent results. Luciferase measurements were repeated across independent cultures and plates; CRISPRi effects were confirmed with ≥2 sgRNAs per target alongside non-targeting controls; STARR-seq results were concordant across independent libraries. Computational analyses were rerun from raw data with version-controlled scripts and yielded identical outputs.
Randomization	For plate-based experiments, constructs/conditions were assigned to wells at random and plate positions were balanced to minimize edge/batch effects; acquisition order was randomized where possible. For CRISPRi, targeting and control sgRNAs were randomly positioned on plates. Randomization is not relevant to purely computational re-analyses of public datasets.
Blinding	Blinding was not applicable during data acquisition for automated plate reader/cytometer workflows.

Reporting for specific materials, systems and methods

We require information from authors about some types of materials, experimental systems and methods used in many studies. Here, indicate whether each material, system or method listed is relevant to your study. If you are not sure if a list item applies to your research, read the appropriate section before selecting a response.

Materials & experimental systems

n/a	Involved in the study
☒	☐ Antibodies
☐	☒ Eukaryotic cell lines
☒	☐ Palaeontology and archaeology
☒	☐ Animals and other organisms
☒	☐ Clinical data
☒	☐ Dual use research of concern
☒	☐ Plants

Methods

n/a	Involved in the study
☒	☐ ChIP-seq
☒	☐ Flow cytometry
☒	☐ MRI-based neuroimaging

Eukaryotic cell lines

Policy information about cell lines and Sex and Gender in Research

Cell line source(s)	Human K-562 (ATCC CCL-243; RRID: CVCL_0004)
Authentication	No, as directly obtained from ATCC.
Mycoplasma contamination	Routinely tested and negative.
Commonly misidentified lines (See ICLAC register)	none

Plants

Seed stocks	*Report on the source of all seed stocks or other plant material used. If applicable, state the seed stock centre and catalogue number. If plant specimens were collected from the field, describe the collection location, date and sampling procedures.*
Novel plant genotypes	*Describe the methods by which all novel plant genotypes were produced. This includes those generated by transgenic approaches, gene editing, chemical/radiation-based mutagenesis and hybridization. For transgenic lines, describe the transformation method, the number of independent lines analyzed and the generation upon which experiments were performed. For gene-edited lines, describe the editor used, the endogenous sequence targeted for editing, the targeting guide RNA sequence (if applicable) and how the editor was applied.*
Authentication	*Describe any authentication procedures for each seed stock used or novel genotype generated. Describe any experiments used to assess the effect of a mutation and, where applicable, how potential secondary effects (e.g. second site T-DNA insertions, mosiacism, off-target gene editing) were examined.*
